# Computer-Aided Ligand Discovery for Estrogen Receptor Alpha

**DOI:** 10.3390/ijms21124193

**Published:** 2020-06-12

**Authors:** Divya Bafna, Fuqiang Ban, Paul S. Rennie, Kriti Singh, Artem Cherkasov

**Affiliations:** Vancouver Prostate Centre, University of British Columbia, 2660 Oak Street, Vancouver, BC V6H 3Z6, Canada; dbafna@prostatecentre.com (D.B.); fban@prostatecentre.com (F.B.); prennie@prostatecentre.com (P.S.R.); ksingh@prostatecentre.com (K.S.)

**Keywords:** estrogen receptor, breast cancer, computer-aided drug design, virtual screening

## Abstract

Breast cancer (BCa) is one of the most predominantly diagnosed cancers in women. Notably, 70% of BCa diagnoses are Estrogen Receptor α positive (ERα+) making it a critical therapeutic target. With that, the two subtypes of ER, ERα and ERβ, have contrasting effects on BCa cells. While ERα promotes cancerous activities, ERβ isoform exhibits inhibitory effects on the same. ER-directed small molecule drug discovery for BCa has provided the FDA approved drugs tamoxifen, toremifene, raloxifene and fulvestrant that all bind to the estrogen binding site of the receptor. These ER-directed inhibitors are non-selective in nature and may eventually induce resistance in BCa cells as well as increase the risk of endometrial cancer development. Thus, there is an urgent need to develop novel drugs with alternative ERα targeting mechanisms that can overcome the limitations of conventional anti-ERα therapies. Several functional sites on ERα, such as Activation Function-2 (AF2), DNA binding domain (DBD), and F-domain, have been recently considered as potential targets in the context of drug research and discovery. In this review, we summarize methods of computer-aided drug design (CADD) that have been employed to analyze and explore potential targetable sites on ERα, discuss recent advancement of ERα inhibitor development, and highlight the potential opportunities and challenges of future ERα-directed drug discovery.

## 1. Introduction

### 1.1. Breast Cancer and Evolution of Its Treatment

Breast cancer (BCa) is the most common lethal cancer in women with projected 279,100 new cases and 42,690 deaths in the United States alone this year [1,2]. Although incidence rates have been increasing, the BCa mortality in North America and the European Union (EU) has decreased. This is attributable mostly to early detection, efficient systemic therapies, and the continual focus on developing better drugs for treatment and prevention of BCa [3]. Early-stage BCa, which is contained in the breast or has only spread to the axillary lymph nodes is considered curable in ~70–80% of patients. Advanced or metastatic disease, however, is currently treatable, but not curable, and the goal of all current therapies is to maintain or improve the quality of life.

On the basis of immunohistochemical expression of prognostic and predictive markers including estrogen receptor (ER), progesterone receptor (PR), human epidermal growth factor receptor 2 (HER2) and the proliferation marker Ki67, clinical practice currently uses a classification of BCa into five subtypes: luminal A-like (ER+, PR+ and HER−), luminal B-like HER2− (ER+, ER and PR expression lower than luminal A-like subtype), luminal B-like HER2+ (ER+, HER2+, ER and PR expression lower than luminal A-like), HER2-enriched non luminal (ER−, PR− and HER2+), and triple negatives (ER−, PR− and HER2−) [4]. HER2+ BCa overexpress growth promoting HER2 protein and is treated with the antibody trastuzumab [5,6,7,8]. Treatment options for triple negative BCa include cisplatin based chemotherapy [9,10]. High levels of Ki67 relate to higher proliferation rate of the cancer cells and is used as an indicator of grade of the cancer [7,11,12]. Altogether, hormone receptor (HR) status, HER2 status and Ki67 levels can be used for streamlined BCa treatment options [4,13,14,15].

**Figure 1:**
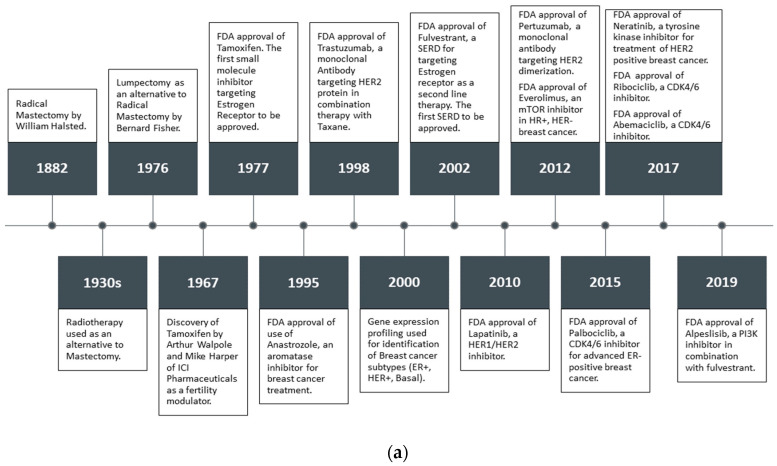

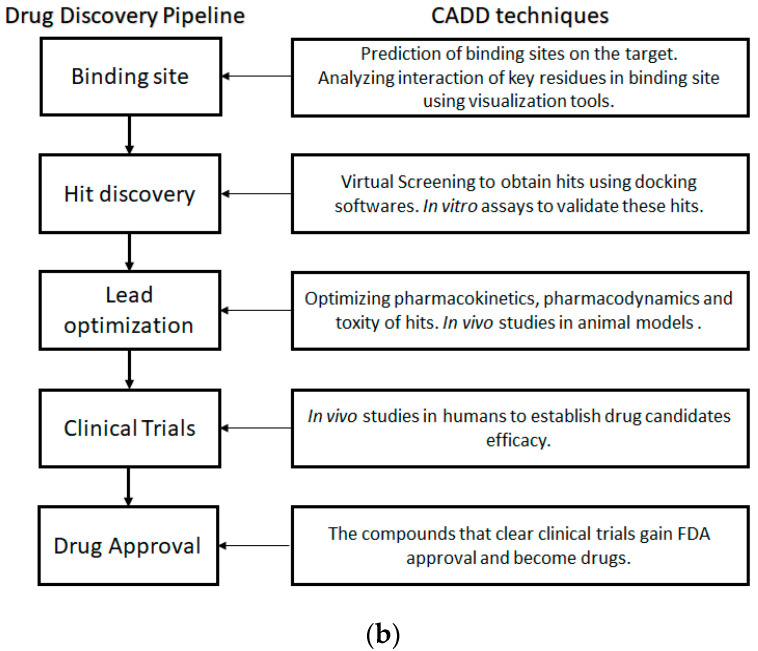
(**a**) Evolution of BCa treatment and therapy; and (**b**) Computer-aided drug design techniques for standard drug discovery pipeline.

Estrogen and progesterone are the primary regulators of breast tissue growth and differentiation. Both steroid hormones are primarily produced in the ovaries in premenopausal women. However, in postmenopausal women, estrogen is predominantly produced by aromatase enzyme activity on androgens [16]. The critical physiological effects of estrogen on growth, development and maintenance of tissues is exerted via the ER. The ERα subtype has also been implicated in BCa pathogenesis and progression [17,18]. More than two–thirds of all BCas are ERα+, and precisely targeting ERα and ERα mediated pathways has been the main strategy in ERα+ BCa treatment and prevention [19]. When BCa cells depend on ERα and or PR for proliferation, they are considered HR+ [20]. FDA approved the selective estrogen receptor modulator (SERM) tamoxifen in 1977, and a selective estrogen receptor degrader (SERD) fulvestrant in 2002 as adjuvant or neoadjuvant hormone therapy (HT) (Figure 1a) with surgery and radiation [21,22,23]. Tremont et al. [24] reviewed the positive clinical outcome of HT, which includes standard treatment with ER antagonists and/or aromatase inhibitors (AI). Treatment with tamoxifen for five years has been the gold standard especially for premenopausal BCa patients. In postmenopausal women, AIs for five years are superior to tamoxifen, and are considered the new standard of care with conflicting data in the literature [25,26]. However, there is no consensus on the ideal sequence of tamoxifen and/or AI and optimal duration of AI therapy.

Combination therapy has been shown to achieve higher efficacy; such as the combination of HT with mTOR inhibitors or CDK4/6 inhibitors are used for tumors with enhanced mTOR signaling and for CDK4/6 sensitive cancer, respectively [27,28,29,30,31,32,33,34,35]. Notably, Harbeck et al. [4] comprehensively reviewed the prognostication and treatment decision- making of different subtypes of BCa. It is broadly accepted that tamoxifen improves survival in BCa patients, reduces the recurrence rate and prevents BCa in high risk women. However, about 40% of the women treated with tamoxifen develop resistance to the drug [36,37].

### 1.2. Computer Aided Drug Design for Estrogen Receptor Inhibitors

Empirical techniques have been conventionally used in ER drug development; and later experimental-based high throughput screening (HTS) played an important role for the identification of potential hits. Recently, computer-aided drug design (CADD) approaches have contributed to processing cheminformatics and bioinformatics information far more efficiently, thereby accelerating early drug discovery efforts through rigorous molecular docking and free energy perturbation simulations [38]. The ever increasing number of X-ray, NMR and Cryo-EM structures has made the structure based drug design (SBDD) process even more reliable and practical [39]. The general drug development workflow with respective stages is featured in Figure 1b, which can be routinely applied to ER drug discovery.

It should be noted that interactions between the binding site and ligand are complex and difficult to understand without intuitive visualization. In recent years, CADD has advanced dramatically with respect to molecular graphics, docking, scoring functions, molecular dynamic simulation, and various machine learning modeling techniques [40,41,42,43,44]. Different CADD tools can significantly accelerate the progression through different drug stages.

In the absence of experimental structures, homology models of the target protein can be built using protein modeling tools if viable templates with significant sequence identity are available, or with de-novo methods if there are no templates to work with [45,46]. Structural elucidation of intrinsically disordered proteins (IDP) can be difficult using traditional structure solving methods. Thus, FRAGFOLD-IDP approach can be used to find the exact stretches in a protein that are disordered and models can be built using the IDP-LZerD algorithm [47,48]. Molecular Dynamics (MD) simulations can simulate the possible native structures of these stretches for SBDD [49].

Availability of X-ray structures of the protein and ligand is extremely valuable for identification and understanding of the binding site for further SBDD. The MOE SiteFinder tool among other software suites such as, PASS, Q-SiteFinder etc. are user friendly tools to examine the protein–ligand interactions and binding site characteristics [42,50,51]. Docking programs such as Glide, ICM, AutoDock Vina and several others have made it possible to screen ligand molecules (taken from existing databases or prepared de-novo) efficiently with pose prediction and ranked list as outputs [43,52,53,54]. Although these lists are not precise, they can be used to cut down small molecule libraries to manageable subsets of compounds to be experimentally tested.

An alternative strategy to develop inhibitors for proteins with no solved structure is to use ligand based drug design (LBDD) approaches. This strategy requires pre-existing activity data of known ligands. Pharmacophore models based on common features of known ligands or the use of Quantitative-Structure activity relationship (SAR) models may be utilized for screening for compounds with desired properties [55,56]. The use of a pseudo-model which involves the building of the binding pocket based on known ligands can also aide drug discovery as it takes into account ligands, their binding poses and solvation scenarios. Thus, the binding site can establish the consistency in binding poses and can be employed using software such as Quasar [57,58]. Lloyd et al. (2004) previously used five widely known antagonists for building de-novo non-steroidal antagonists using SKELGEN without the use of target structure [57,59]. Pharmacophore models and Quantitative-SAR may also facilitate lead optimization on already known hits with ADMET predictors and FEP+ for suggesting better hits [38,55,56].

Continued efforts in building and curating benchmarking datasets along with large scale initiatives such as Environmental protection Agency’s (EPA’s) endocrine disruptor program have also been immensely useful for evaluating performances of docking software and training predictive machine learning models for major nuclear receptors including ER.

In this review, we present the recent advancements in ERα inhibitor development and highlight the potential opportunities and challenges of future ERα-directed small molecule inhibitor development.

## 2. Estrogen Signaling Pathway

### 2.1. ER Gene Expression. Transcriptional, Epigenetic and Post-Transcriptional Control

**Figure 2:**
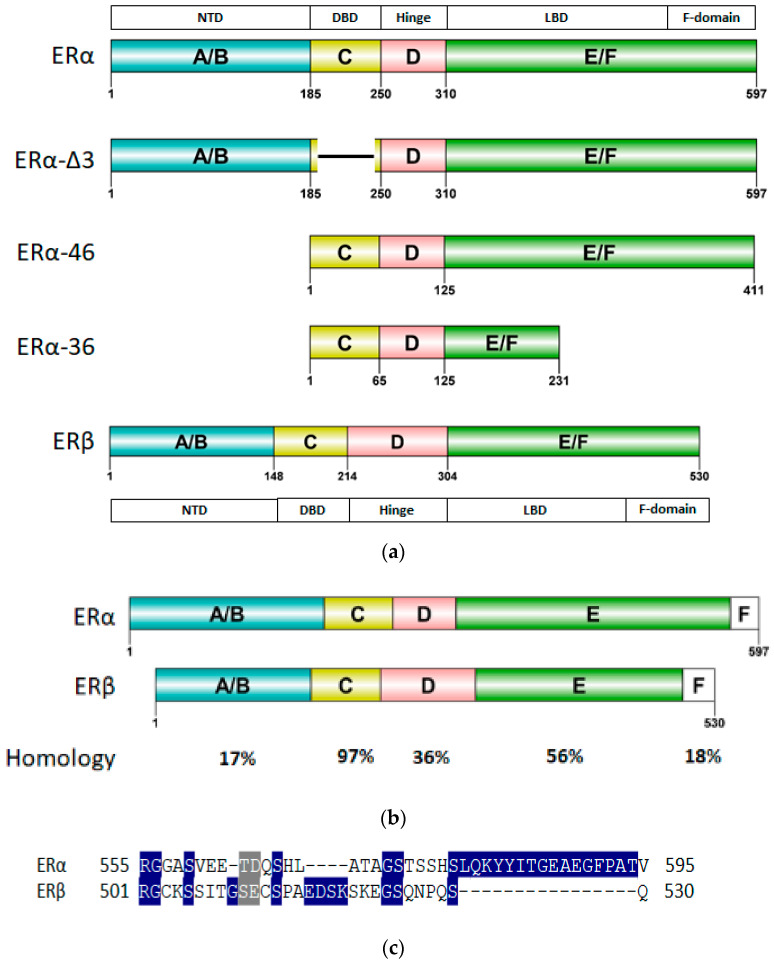
(**a**) ERα and ERβ domain organization; (**b**) homology of ERα and ERβ at different domains; and (**c**) F-domain sequence alignment between ERα and ERβ.

The two subtypes of ER, ERα and ERβ, are made up of eight exons each and are encoded by two different genes (Figure 2). The 140-kB-long *ESR1* gene located on the long arm of chromosome 6 at q25.1 encodes for ERα, a 66.2-kDa protein while the *ESR2* gene on the long arm of chromosome 14 at q23.2 encodes the 59.2-kDa ERβ protein. Nine *ESR1* gene promoters have been identified so far. These promoters act as a binding site for multiple transcription factors (TFs) that may play a role in tissue and functional specificity. TFs such as AP2a, AP2g, ERBF-1, Foxo3a, FoxM1 and GATA-3 positively regulate *ESR1* gene expression while some TFs such as Blimp-1 act as *ESR1* expression repressor [60,61]. These TFs may be tissue specific and their expressions related to BCa induction. Thus, NK-kB can induce Blimp-1 and Enhancer of Zeste Homolog2 expression which, in turn, may inhibit *ESR1* expression. However, it can directly induce ERα transcriptional activity [61]. *ESR1* gene amplification has also been implicated in ERα regulation and found frequently in BCa patients [62,63]. Two *ESR2* gene promoters have been identified, *0K* and *0N*, that bind various TFs such as c-jun, AP2a, AP2g, CREB and Clock/BMAL1 that positively promote *ESR2* transcription [60,61].

Notably, CpG island methylation at the ERα promoter has been linked to ERα- BCa, development of aggressiveness, malignancy and resistance to HT [60,64]. Such methylation can directly block the recruitment of TFs to their corresponding binding sites or change the chromatin structure in inhibitory manner. Demethylating agent treatment or inhibition of methylating agents (such as DNA methylation 1) can re-induce ERα expression in ERα^-^ BCa cells while methyl transferases upregulate ERα transcription [65]. The 0N promoter for ERβ has been found to be methylated in certain cancer tissues including BCa while *0K* promoter was found to be demethylated making *0N* methylation a target for ERβ expression manipulation [61]. Acetylation, methylation or phosphorylation of histones can extensively regulate *ER* gene transcription through chromatin remodeling. Treatment of cells with histone deacetylase inhibitors can induce ERα transcription in ERα^-^ BCa cells, rendering the cells sensitive to HT [66].

Post transcriptional regulation of ERα also plays a major role in regulating its expression. AU-rich regions in ERα regulatory regions make it unstable while AUFp45, which protects the mRNA from RNAses, stabilizes the mRNA [61]. Several miRNAs such as miRNA-206, -22, -130a, -17/92 and -145 inhibit ERα expression and in turn miRNA-206, -22, -221 and -222 are inhibited by ERα activity. Although not a lot has been uncovered about miRNA activity on ERβ, it was found that miRNA-92 downregulates ERβ expression [61].

### 2.2. ER Protein Organization. Structural Aspects of ER Interaction

ER is a member of the five-membered steroid hormone receptor (SHR) subfamily which includes Androgen Receptor (AR), Progesterone Receptor (PR), Glucocorticoid Receptor (GR) and Mineralocorticoid Receptor (MR). The two isoforms of ER have the same characteristic domain organization of SHR [67]. Remarkably, ERα and ERβ can have opposing effects at the promoters of genes involved with proliferation with ERα having a driving effect while ERβ an inhibitory effect on cell proliferation [61,68,69,70,71].

ER, similar to other SHRs, consists of six functional domains labeled A–F: The N-Terminal Domain (NTD) (A/B, encoded by exon 1), the DNA binding domain (DBD) (C, encoded by exons 2–4), the hinge region (D) encoded by exon 4 with the ligand binding domain (LBD) (E) and the C-terminal domain (F) encoded by exons 5–8 as shown in Figure 2a. Since the homology between ERα and ERβ differs for the consistent domains, some receptors’ regions can therefore be strategically used for selective targeting of ERα (Figure 2b).

The ER-NTD section houses the important activation function −1 (AF1) which is essential for ER transcriptional activity [72]. Post translational modifications in this area have been shown to result in ligand independent signaling of ER by direct co-regulatory protein recruitment [73,74,75,76]. This region is very poorly conserved between all the SHRs with merely 17% homology between ERα and ERβ. No X-ray structure of ER-NTD has been solved yet as the region is flexible and intrinsically disordered [73]. It has been found to assume a folded, more orderly state when in contact with certain co-regulators [77]. It has been suggested that AF1 of ERβ may have inhibitory effects on cell proliferation as it has been shown to lack the binding ability to SRC-1, a co-regulatory protein [78].

The highly conserved across all SHRs, DBD region (with a sequence identity of 97% between ERα and ERβ), consists of two Zinc finger motifs recruited for the DNA binding. The first zinc finger encompasses a proximal-box or the P-box which is responsible for recognition of Estrogen Response Element (ERE) on the DNA strand [79,80]. The second zinc finger contains the distal-box or the D-box which recognizes the spacing between palindromic ERE and forms the interface for ERE aided ER-DBD dimerization [79,80]. There is a stretch of amino acids at the C-terminal of the ER-DBD that is flexible and involved with DNA binding [81]. However, generally, structural details of ER-DBD are somewhat sparse, as only one NMR structure of the DBD monomer and one of ER-DBD dimer in complex with ERE-DNA has been solved so far [82,83].

The hinge region is highly variable amongst SHRs with a low sequence identity (36%) between ERα and its subtype, ERβ. This region has a flexible C-terminal and has been implicated in nuclear localization signaling and co-regulatory protein recruitment by synergizing AF1 and Activation Function-2 (AF2) function in ERα [7,78,84]. There are four X-ray crystal structures and two NMR structures reported for the hinge region. Five out of the six available structures cover the region near the C-terminus of the hinge domain bordering the ER-LBD [85,86,87].

**Figure 3:**
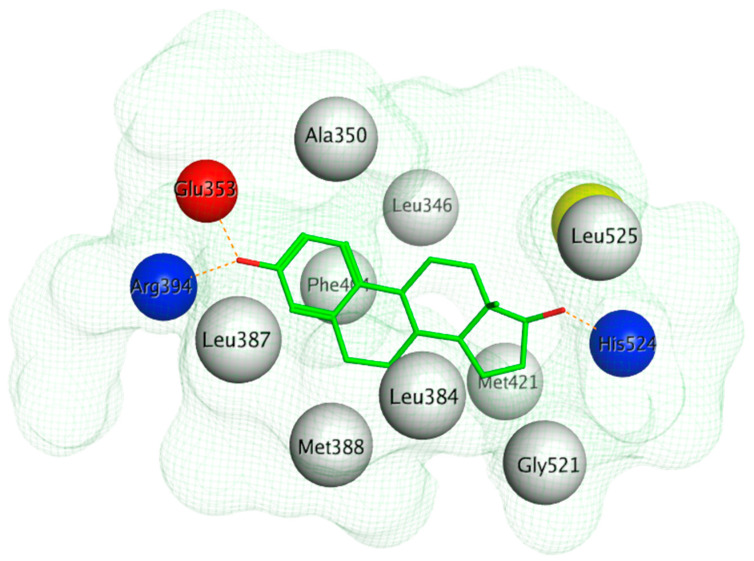
E2 (Green) bound to wild-type ERα at the EBS (pdb: 1QKU). The hydrogen bonds are depicted using dashed lines (orange). E2 forms two hydrogen bonds on one side with Arg394 and Glu353 and forms one on the other side with His524.

The ERα-LBD has been extensively studied with approximately 265 X-ray structures, some of which include the C-terminal segment of the hinge region. There are 36 X-ray structures of ERβ-LBD reported so far. Although this region shows only 57% identity with ER-LBD their overall structures and mechanism of action are similar. The ER-LBD houses the estrogen binding site (EBS), AF-2 site and the ER-LBD dimerization interface. It consists of 12 α-helices (H1–H12) forming three layers with H4–6, H8 and H9 sandwiched between H1 and H3 on one side and H7, H10 and H11 on the other (Figure 5) [88]. This fold forms a highly hydrophobic cavity corresponding to the EBS. The use of MOE package enabled visualization of critical protein–ligand interaction presented in Figure 3, where in native conditions, E2 binds to the hydrophobic cavity formed between the sandwiched helices of ERα comprising of the residues Met343, Leu346, Ala350, Glu353, Leu384, Leu387, Met388, Leu391, Arg394, Phe404, Met421, Ile242, Leu428, Gly521, His524 and Leu525 [42,89]. The hydroxyl group on the A ring/phenolic ring of E2 forms H-bonds interaction with Glu353 and Arg394 while the hydroxyl group on the other end of the compound interacts with the Sulfur in Met343 [89].

Binding of estrogens to the EBS causes a conformational change in the receptor due to the movement of H12 which, in turn, opens up the AF-2 site for coactivator recruitment [17,90]. AF1 and AF2 act in synergy for full transcriptional activity of ER [72]. Furthermore, H8–H11 motifs play a role in LDB dimerization with most important interactions corresponding to H10 and H11 [91,92]. These helices are also implicated in hormone binding interactions. It has also been demonstrated that ER-LBD can form both homo and heterodimers [93]. H11 is also important because it contains the nuclear localization signal [94]. The amplification of signals in the ER pathway leads to uncontrollable cell proliferation and growth resulting in tumor formation [95].

The F-domain can be promising for introducing the desired selectivity in ER inhibition as it is highly variable amongst all the SHRs and is poorly conserved in the two subtypes of ER (18% identity as shown in Figure 2c). It has been implicated in ligand-based regulation of ER mediated transcription [96,97]. The F-domain was also found to be involved in inhibition of LBD dimerization, tamoxifen responsiveness and antiestrogen mediated transactivation of ERα [96,98]. Thus far, eight structures of C-terminal segments of F-domain in complex with 14-3-3 protein have been experimentally resolved [99,100].

Wild type ERα has three known naturally occurring variants: ERα-46, ERα-36 and ERα-delta3 (Figure 2a). ERα-46 has a truncated AF1 that is involved in hormone dependent growth of cancer cells and its induction in tamoxifen resistant cells restores sensitivity [101,102,103]. Another ERα variant, ERα-36, with a spliced AF1 and a portion of LBD, regulates hormone dependent and independent signal transduction in cancer cells and increases the tumorigenesis and invasiveness [104,105,106]. ERα-36 has been reported to induce sensitivity to ERα antagonists [107]. ERα-delta3 variant of ERα with a truncated DBD, reduces metastasis and proliferation in BCa cells [108,109].

Point mutations in *ESR1*, have been reported play a part in HT resistance. Thus, frequent point mutations have been found in HT relapsed patients with metastatic BCa, but not in untreated patients. These mutated cells may evade the initial round of HT with *ERS1* mutation found in 22% of the metastatic BCa patients [110,111]. Residues 534–538 have been the hotspot of mutations with additional mutations discovered on Ser463 and Glu380 [111]. Moreover, mutations such as L536N, Y537S, Y537N and D538G may activate ERα in the absence of an agonist [112,113,114,115]. Current clinical dosage of antagonists are ineffective on mutant forms of ERα. Higher dosages of the same have been shown to be effective on some mutants [109].

### 2.3. Estrogens

Estrogens are female sex hormones majorly produced in the ovaries and in the placenta (during pregnancy). Estrogen steroids play a paramount role in the development, regulation and maintenance of female reproductive system [116,117]; they are essential in cognitive health, metabolism, bone formation, mobilization of neutrophils and cardiovascular activity among others [116,118,119,120,121,122,123].

Primarily, androgen synthesis from cholesterol occurs in the thecal cells of ovaries and aromatization of these androgens in granulosa cells leads to the production of estrogens but they can also be produced by other non-gonadal sites such as brain, adipose tissue, bones, liver, adrenal gland, skin and blood vessels [116,124]. Estrogen formation is tightly regulated by the hypothalamic pituitary ovarian axis [125,126]. Gonadotropin-releasing hormone stimulates the anterior pituitary gland to release two hormones: luteinizing hormone, which initiates the synthesis of testosterone, and follicle-stimulating hormone, which regulates the expression of aromatase cytochrome P450 [125]. Cytochrome P450 aromatizes androgens to produce estrogens. Follicle-stimulating hormone is the rate limiting step in estrogen production [127].

There are four naturally occurring types of estrogen: estrone (E1), 17β-estradiol (E2), estriol (E3) and esterol (E4). E1 and E2 are produced mainly by the ovaries, E3 is produced by the placenta and E4 is produced primarily in the fetal liver during pregnancy [125,128]. E2 is the predominantly produced estrogen and the most potent of them all, being 100-fold more potent than E3 and 10 times more potent than E1 [129].

### 2.4. ER Transcription Mechanism

**Figure 4:**
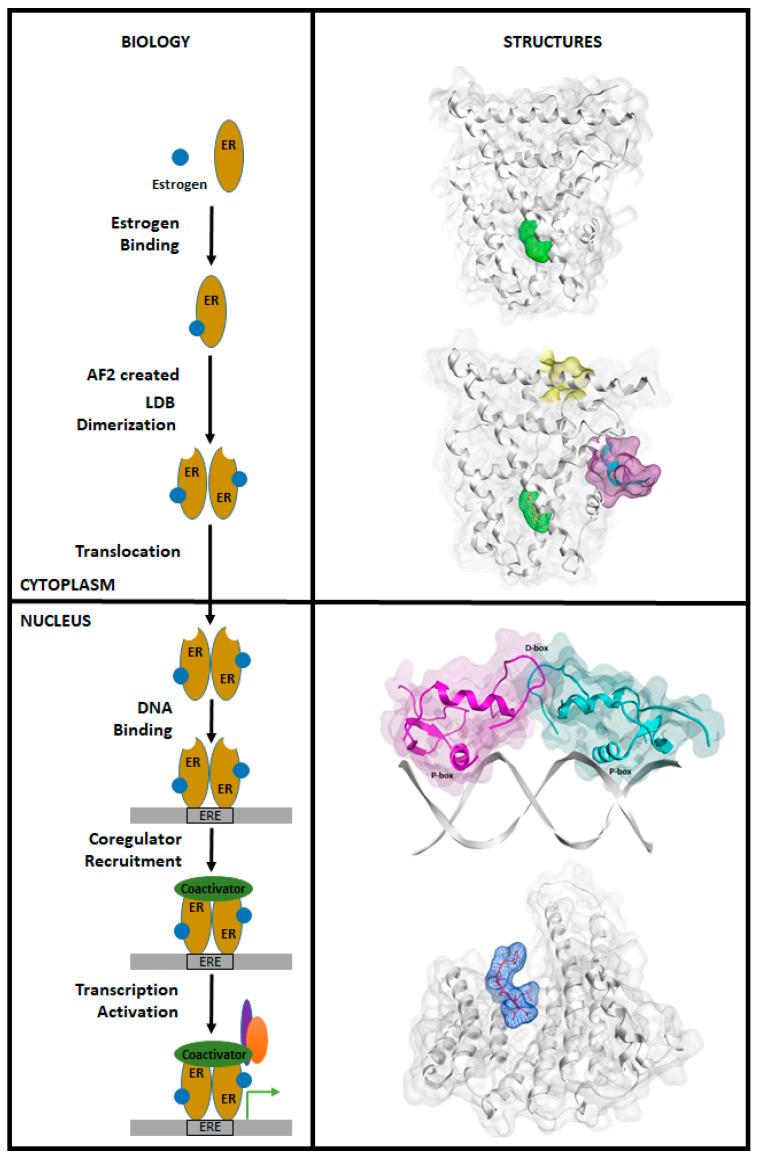
The biology of classic ER transcriptional mechanism (left) with the corresponding structures (right): the first structure depicts E2 (green) bound to the ER-LBD; the second structure depicts an E2 (green) activated ER with an coactivator docked to the created AF-2 site (purple) along with the potential dynamic BF3 site (yellow); the third structure shows a translocated DBD/DBD ER dimer bound to DNA; and the fourth structure shows the F-domain bound to an AF-1 mediated transcription repressor protein, 14-3-3 (white).

The classic ER transcription mechanism is shown in Figure 4 (left). The binding of E2 at the EBS site featured in Figure 4 (right) triggers the signaling pathway by activating cytoplasmic ER with a conformational change that subsequently triggers receptor dimerization, nuclear translocation and exposes the ER-AF2 site for subsequent co-activator binding [130,131,132,133,134]. Once in the nucleus, the DBD region of the ER recognizes and interacts with the ERE, a consensus 5′-GGTCAnnnTGACC-3′ palindromic sequence, for nuclear transcription as a DBD/DBD dimer and then the ER complexes with co-activators such as p160 family of proteins that includes Steroid receptor Coactivator (SRC-1), GRIP-1 and AIb1 (Figure 4) [135]. In addition, the F-domain, when not in contact with repressor proteins such as 14-3-3, interacts with AF1 for full transcriptional activity of ER [99]. It is of particular importance that all these consequent ER activation steps could, in principle, provide significant opportunities for modulating ER functioning with small molecules.

## 3. Small Molecule Inhibitors

### 3.1. Targeting EBS

#### 3.1.1. Selective Estrogen Receptor Modulators (SERMs)

SERMs can exert agonistic or antagonistic effects on ER depending on the tissue type, receptor’s subtype and the ERE promoter sequences. As antagonists, SERMs induce a conformational change in ER to its inactive state [136,137,138]. The most notable SERMS are collected into Table 1, and their brief historical background is presented below.

In the 1960s, in pursuit of developing a potential non-steroidal anti-estrogen contraceptive using a triphenylethylene core, Dr. A. L. Walpole at ICI Pharmaceuticals (Astra Zeneca) discovered a compound ICI146,474, later known as tamoxifen. This first FDA-approved SERM has become the gold standard drug for ERα+ BCa treatment and prevention in both pre- and post-menopausal women, and since 1978 it has paved the way for further generations of SERMs [139,140,141]. Five-year adjuvant therapy with tamoxifen results in a 75% decrease in risk of recurrence between 10 and 14 years and 80% with a 10-year administration in ERα+ patients [142]. In treatment of pre-menopausal women with advanced BCa, the response rates ranged from 20% to 45%, while for ERα+ and ERα+/PR + cancers response rates were 50% and 60–70%, respectively, for post-menopausal women with advanced BCa [143]. However, long-term administration of tamoxifen results in an acquired drug resistance in the initially responsive tumor [144]. Resistance may be caused by cells expressing certain regulators interacting with tamoxifen-bound ER [36,37], crosstalk between HER2 and ERα or ligand independent signaling through other pathways of PI3K/mTOR or NFkB [145,146,147], differential microRNA expression [145,148,149,150,151] or increase in E-cadherin methylation [152], among other mechanisms reviewed elsewhere [153,154,155]. In addition, tamoxifen has an agonistic effect on endometrial cells leading to an increased risk of developing uterine cancer. Moreover, it causes undesired pure antagonist effects on ERβ [156].

**Figure 5:**
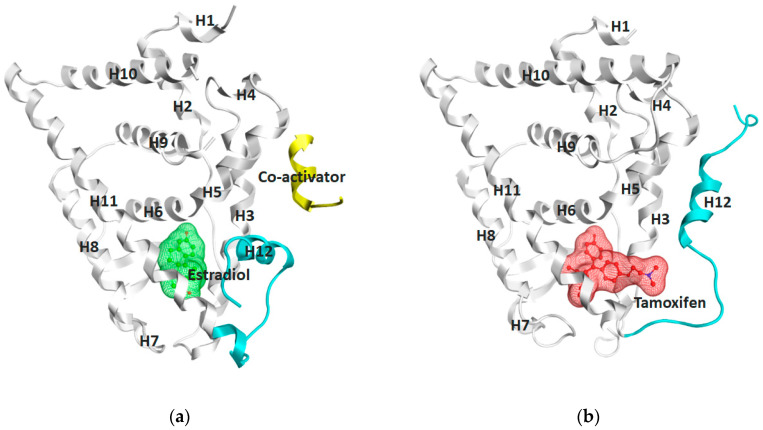
(**a**) E2 (green) bound to ERα (white) and the coactivator peptide (yellow) (pdb: 3UUD) with the helix numbers from 1 to 12 specified. The binding of E2 moves the H12 to its active conformation, creating the AF2 site that binds to Co-activator proteins. (**b**) OHT (red) bound ERα (white) (pdb: 3ERT). Binding of OHT moves the H12 to an antagonistic conformation, blocking the AF2 site.

Comparison of E2 and 4-hydroxy-tamoxifen (OHT), a metabolite of tamoxifen, bound to ERα illustrates the structural transformation that leads to the antagonist action of OHT (Figure 5).

The desired therapeutic effect of OHT is exerted by a structurally deactivated ER-LBD. When E2 binds to the highly hydrophobic EBS, the ER is activated through coactivator binding (yellow) to the AF2 site dynamically created due to the H12 orientation (cyan) represented in Figure 5a. However, upon OHT binding, the H12 (cyan) is repositioned (schematically shown in Figure 5b) such that it blocks the AF2 site required for coactivator binding. The consequence of the conformational change of H12 in ER-LBD results in the antagonist action of OHT as can clearly be seen in Figure 5 visualized using MOE [42,157]. In 2016, Ho Leung Ng ran a 240-ns MD simulation on both E2 bound ERα (pdb entry 1ERE) and OHT bound ERα (pdb entry 3ERT) to find that not only did the OHT/antagonist bound ERα display disorder in both H12 edges and undergo fluctuations but it also resulted in a stabilized antagonistic conformation of H12 [158]. MD simulation has also been previously used to study the differences between ERα and ERβ for ligand selectivity [159].

**Figure 6:**
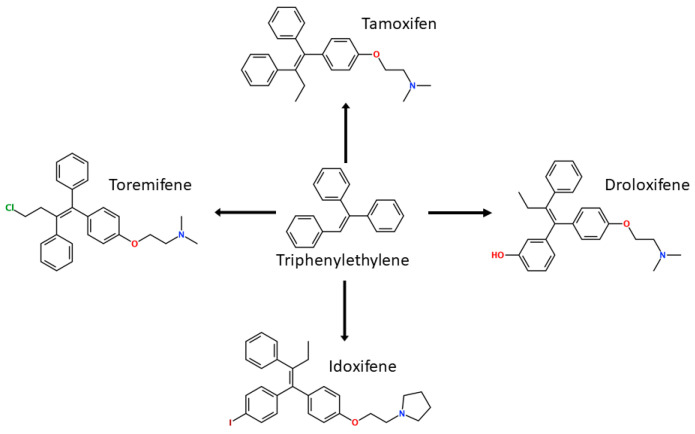
Common triphenylethylene core used to build tamoxifen, toremifene, droloxifene and idoxifene.

Following the success of tamoxifen in BCa suppression, new compounds with either the same or different cores were developed. Thus, to further optimize tamoxifen, its close derivatives toremifene, droloxifene and idoxifene were synthesized, all carrying the core triphenylethylene structure (Figure 6) [160,161].

Tamoxifen was the only approved anti-estrogen for BCa treatment until 1997, when toremifene also passed FDA approval [162,163]. It demonstrated similar tolerability and safety as tamoxifen and its long-term administration also elevated the risk of endometrial cancer development [164]. On the other hand, while toremifene was found to be three times less potent than tamoxifen, it was characterized as less carcinogenic compared to tamoxifen. More importantly, toremifene is more selective towards ERα than ERβ, while tamoxifen is nonselective [165,166]. Of note, both droloxifene and idoxifene were never marketed, as they demonstrated lesser efficacy and response rate compared to tamoxifen, and were terminated in the respective Phase III trials [167,168]. All three of these molecules were found to be cross-resistant towards tamoxifen in the corresponding resistant cells [167,168,169].

To eliminate the risk of endometrial cancer development and cross-resistance to tamoxifen in first generation SERMs, further pharmaceutical development led to second and third generation SERMs. Thus, raloxifene, a benzothiophene-based short-acting drug, is the only established second generation SERM [170]. Initially, Black and Goode (1980) and Jones et al. (1983) noted the effects of raloxifene on BCa cell lines and mammary tumors in rats, respectively [171,172]. It failed initially in a Phase II trial in metastatic BCa for lacking activity [173], but was later approved in 2007 for BCa prevention in postmenopausal women with high risk. The IC_50_ values of raloxifene in proliferation inhibition of MCF-7 cells were found to be 0.4 ± 0.3 nM [174]. In addition, it was found to reduce uterine cancer development by almost half compared to tamoxifen, though it showed lower efficacy than tamoxifen [138,166]. However, results from a randomized trial concluded that raloxifene increases the risk of thromboembolism and strokes in post-menopausal women [175,176]. Moreover, due to its poor solubility and metabolic instability, it has poor bioavailability (2%) [177].

To address the shortcomings of these two drugs, the third generation of SERMs including arzoxifene and others with improved bioavailability were developed (Table 1). Palkowitz et al., in 1997, substituted the carbonyl group in raloxifene with an oxygen atom to make arzoxifene using SAR modeling [174]. IC_50_ of arzoxifene in inhibiting MCF-7 cell proliferation is 0.05 ± 0.02 nM, which is significantly more potent than raloxifene [174]. Arzoxifene displayed cross-resistance to tamoxifen in MCF-7 cells but not in T47D cells [178]. It has lower risk for development of endometrial cancer than tamoxifen, but also has shorter time to treatment failure than tamoxifen and it produces half the median progression free survival of tamoxifen, which resulted in the failure of its Phase III trial [179,180].

Other third generation SERMs, bazedoxifene and lasofoxifene (both developed by Pfizer), have been approved for the treatment of osteoporosis. Bazedoxifene is a highly potent indole-based SERM/SERD hybrid compound, which was found to have an IC_50_ of 0.19 nM in inhibiting E2 mediated proliferation of MCF-7 cells [181,182]. Bazedoxifene displays SERD like activity but acts predominantly as a SERM in BCa tissue [181,183]. It binds to ERα with an IC_50_ of 26 nM and has shown activity on tamoxifen-resistant cells [181,183]. It displays a slightly higher affinity towards ERα than ERβ with reported IC_50s_ of 14 and 40 nM, respectively, as measured using a radioligand binding assay [165,184]. In 2009, the drug was approved as a monotherapeutic agent for the prevention and treatment of osteoporosis in post-menopausal women in the EU. Since then, the drug has also been FDA-approved as a combination therapy with conjugated estrogens for prevention of osteoporosis in postmenopausal women [185]. Phase Ib/II study of bazedoxifene administered in combination with palbociclib (a CDK4/6 inhibitor) in pre-treated HR+ BCa concluded that the combination is tolerable and displayed notable activity [186]. The drug is currently in a Phase II trial for in-situ ductal carcinoma patients for determining its effect on proliferation markers of BCa [187].

Lasofoxifene is a tetrahydronaphthalene based SERM marketed under the name of Fablyn. It is an oral drug that has been shown to decrease ERα+ BCa risk with higher inhibitory activity than tamoxifen [165,188]. Lasofoxifene has an IC_50_ of 1.08 nM towards ERα and 4.41 nM for ERβ. Lasofoxifene treatment for five years leads to endometrial changes but does not pose uterine cancer development risk [189]. There is currently an ongoing Phase II trial to study the activity of lasofoxifene against fulvestrant in *ESR1* mutated advanced/metastatic ERα+/HER− BCa [190].

**Figure 7:**
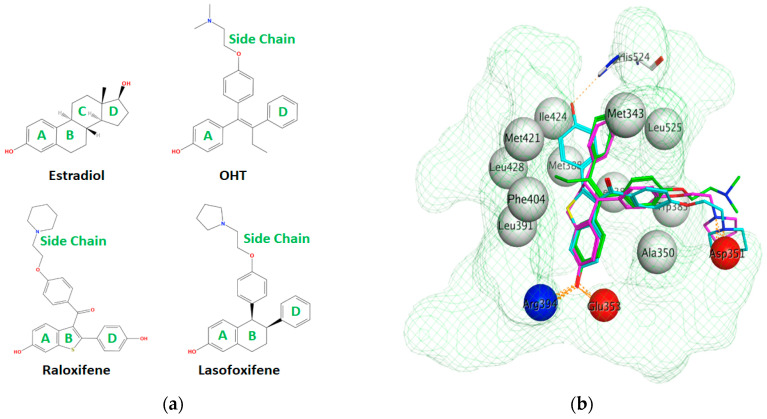
(**a**) Ring nomenclature and side chain of OHT, raloxifene and lasofoxifene in accordance with conventional E2 nomenclature; and (**b**) OHT (green), raloxifene (cyan) and lasofoxifene (pink) complex with the EBS (pdb: 3ERT, 1ERR and 2OUZ) with dashed lines (Orange) depicting hydrogen bonds.

Using MOE visualizing package, we can see that raloxifene and lasofoxifene bind to the EBS in the same manner as OHT (Figure 7) [42,92,191,192]. The structure analysis demonstrates that the A-ring hydroxyl group of all ligands forms H-bonds with the Glu353 and Arg394. The D-ring phenolic hydroxyl group of raloxifene interacts with a rotated His524 sidechain which is flexible in nature [193]. The OHT side chain is stabilized by a salt bridge formation between the dimethylamino group on the side chain and Asp351 [191]. The piperazine ring nitrogen in the side chain of raloxifene and pyrrolodine nitrogen in the side chain of lasofoxifene interact with Asp351 [92,192]. As shown in Figure 5, raloxifene and lasofoxifene similar to OHT displace the H12 (cyan in Figure 5) due to steric clashes of their bulky side chains with EBS residues leading to inactive conformation of ER [92,191,192,194].

Many small molecules have recently been claimed to be fourth generation SERMs due to their improved activity, but only acolbifene, a benzopyran derivative discovered by Labrie et al. in 1999, is well established [195]. EM-800 is a well-studied precursor of acolbifene which was found to be safe and tolerable [196,197]. It is orally active but lacks agonistic activity in the endometrium, thereby reducing the chances of developing uterine cancer [198,199,200]. The drug was found to have partial cross-resistance with tamoxifen [198]. Acolbifene is currently in Phase II trial for its activity in premenopausal women who are at high risk for developing BCa [199].

Readily available structures of ER-LBD along with molecular docking programs has made it easier to study the docked poses of empirically bound ligands. A few authors have used docking to study the poses and conformations of these compounds and have found them to be potent in vivo and/or in vitro experiments or to predict their relative activity. In 2019, Sharma and colleagues used in silico docking to support the activity of their designed and synthesized tetrahydro-b-carboline–isatin conjugates with both tetrahydro-b-carboline and istatin C-1 and C-5 stereomers [201]. The most promising hit displayed an IC_50_ of 37.42 µM against MCF-7 cells as opposed to 50 µM for tamoxifen and had minimal effects on ERα- MDA-MB-231 cells thus confirming that their actions are ERα mediated [201]. The authors used Chem3D Ultra to draw their ligands and Avogadro1.2.0 tool to optimize them for docking to the UCSF Chimera prepared protein (pdb entry 3ERT) using AutoDock Vina [54,201,202,203]. The molecule had similar hydrophobic interactions to tamoxifen and scored fairly high in terms of docking [201]. All the conjugates were predicted to bind better than tamoxifen, but this was not the case experimentally as docking scores are not always representative of reality [201]. Similarly, Katzenellenbogen and collaborators used docking to study the poses and contribution of side chains in antagonistic activity of their proposed compounds on mutant forms of ERα [204]; Singla and colleagues used docking to study the poses of experimentally active novel indole-based antagonists [205]; and Lou et al. used AutoDock to model their best designed hits [206,207].

SBDD using docking programs and pharmacophore models have accelerated the drug discovery process. Various groups over the years have employed these CADD tools for finding novel inhibitors from available databases and experimentally testing the best hits to validate their findings. Virtual Screening (VS) studies have been conducted for the discovery of ligands for the ERα-EBS pocket [208,209,210,211,212,213,214,215] and for all subtypes of ER [209,210,216,217,218,219,220,221,222].

In 2015, Istyastono and group used PLANTS1.2 software for repurposing of a popular cyclooxygenase-2 inhibitor, celecoxib (Table 2) for ERα (with and without conserved water molecules) [223,224]. After docking, they used these results to find their protein–ligand interaction fingerprints (PLIF) [223]. From the three docking runs, best docking poses and poses with best Tanimoto coefficient (Tc) with respect to the reference OHT fingerprints Tc were selected, and then this method was repeated 1000 times for statistically validating the Tc values [223]. Using the Wilcoxon test, the authors were able to determine that the values were equal or better than the set standard threshold of 0.720, thus confirming that celecoxib may be repurposed as potential ERα ligand [223]. Celecoxib from the marketed drug Celebrex in MCF-7 cell cytotoxicity assay displayed an IC_50_ value of 94.06 ± 14.03 µM compared to that of 40.78 ± 0.48 µM for the reference, tamoxifen giving comparable results [223].

In a similar study, Niinivehmas et al. (2016) used two separate databases—one from ChEMBL and the other from directory of useful decoys (DUD), prepared using LigPrep2.5 and protonated at pH 7.4 using Confgen in force field MMFF94 [225]. Pharmacophore model was built using PHASE3.3 and a 3D-QSAR model was constructed [225,226]. Pdb entry 3ERT (OHT bound to ERα) was prepared using Maestro9.2 with preservation of water in the binding site and then minimized in force field OPLS-2005 [225,227]. Both the datasets were then docked to the prepared structure using Glide5.7 SP and XP as well as PLANTS [43,224]. Negative image based models were created with receptor flexibility taken into account by running MD simulations and then screened across the two databases using SHAEP [225,228]. The authors then used SPECS database for finding hits based on the above models with pharmacophore posing difficulties as five features posed to be too specific and four features were unable to differentiate between the inactives and actives yielding five hits with “Compound S4” (Table 2) showing an IC_50_ value of 0.25 µM in Fluorescence Polarization (FP) assay [225]. A database of coumarin-based compounds was also screened using the same pipeline and the top five hits were synthesized and tested in vitro for IC_50_ values of 0.31–3.1 µM (FP assay) [225].

Pang et al., in 2018, used CADD for the development of antagonists of ERα using the antagonists form BindingDB database and DUD enhanced (DUD-E) decoys, which were further processed with MOE, also adding a marker of 1 for antagonists and −1 for decoys [42,229]. The authors used 2D and 3D descriptors generated using MOE and Discovery Studio selected on the basis of Pearson’s correlation as features for their Naïve Bayesian and Recursive Partitioning Classifier models for fivefold cross validation runs with the best models obtained using MOE 2D descriptors and molecular fingerprints for both model type [42,229,230]. Receptor structure from pdb entry 3ERT was used for molecular docking using LibDock and CDOCKER to further analyze results by the best machine learning models [229,230,231,232]. The best machine learning models along with molecular docking were then used to screen through their in-house database of natural compounds [229]. FP assay was used to analyze their in silico hits with eight compounds, including genistein, displaying antagonism towards ERα within the range of 29.38–977 nM [229].

In same year, Wang et al. assessed cyclopropyl derivatives to develop ERα selective novel SERMs with the aid of docking using Discovery Studio 2.5 against both ERα (pdb entry 1A52) and ERβ(pdb entry 3OLS) [230,233]. The authors were able to discover five compounds with ERα activity (IC_50_ ranging between 1.79–6.27 µM) but with non-detectable binding to ERβ using FP assays [233]. Luciferase reporter Assay confirmed that all the molecules were antagonistic towards ERα and were antiproferative in MCF-7 cells [233]. In a similar study, Jin and group used AutoDock Vina4.0 to dock Benzofuran derivatives in an attempt to identify novel SERMs using the pdb entry 3ERT as the target protein for docking [54,234]. The hits were tested in MCF-7 cells, MDA-MB-231 cells and HEK-293 cells giving IC_50_ value comparable to that of tamoxifen and raloxifene [234].

Hendy et al. created an indole base library and docked the same to protein obtained from pdb entry 1ERE with conformation generated using Omega and docked using FRED [213,235,236]. The compounds had similar interactions as that of E2 along with indole ring interactions with Thr347 [213]. YMA-005 and YMA-006 (Table 2) showed IC_50_ values of 1.76 and 3.31 nM, respectively, in ERα ELISA binding assays [213]. The compounds also inhibited proliferation in MCF-7 and T-47D cell lines with IC_50_ values of around 28.23–32.96 µM, which is lower than that of 34.42 ± 0.83 and 42.40 ± 0.78 µM observed in the case of tamoxifen treatment [213]. Both compounds could reduce tumor size combined with reduction in immunohistochemical expression of ERα [213]. The compounds also caused ERα degradation along with an increase in cell necrosis [213].

Pavlin et al., in 2019, employed computational tools to determine the effects of Y357S mutation on ERα along with the antagonists’ effects on the said mutant and to find novel inhibitors against them [237]. Using Ligfilter (Schrodinger Software), the authors filtered the NCI database (~265,242 compounds) based on Lipinski’s Rule and 10 rotatable bonds and further by logP values that were calculated using QikProp (Schrodinger Software) to improve oral availability of ERα antagonists [227,237]. After running restricted and unrestricted MD using GROMACS5.0.4 to see the behavior of ER mutants Y537S, Y537N and D538G in complex with Endoxifen, AZD-9496 and fulvestrant [44,237]. These models were then docked with the filtered ligand set using Glide HTVS [237]. Top 10% of the hits generated were docked using Glide SP to select top 10% of the generated set for subsequent docking using Glide XP [43,237]. The compounds were selected on the basis of their GlideScore and a consensus based on whether the compound docked desirably to at least one of the five structures generated previously [237]. The authors also employed the CANVAS protocol that is based on scaffolds defined by them for antagonism [237]. The compounds were screened using Glide XP and selected on the basis of their score and whether they displayed desirable interactions with at least two of the five mutant MD structures [43,237]. The selected compounds were experimentally validated using immunoblot analysis, ERα transactivation assay and MTT cell viability assays with a substance called “Compound 19” (Table 2) being the best hit [237]. “Compound 19” could target both wild type and Y537S ERα activity but only at high concentrations [237].

Experimental validation is ultimately needed to confirm the activities of the proposed novel inhibitors as in silico methods lack the ability to completely capture the actions of a ligand in an actual system. However, various simulation packages can help with such predictions. Many groups have employed CADD-based VS but their results remain to be validated experimentally. For instance, in 2012, Istyastono et al. used PLANTS1.2 and Monte Carlo simulations to screen through the compound eugenol with its analogs and dimers [224,238]. In that study, 4-(4-hydroxy-3-(prop-2-en-1-yl)phenyl)-2-(prop-2-en-1-yl)phenol displayed higher ligand efficiency values making it a target for further research and development of a novel ER inhibitor but these claims lacked experimental validation [238]. Likewise, in 2015, they utilized SBDD to identify eugenol analogs as ERα ligands [239]. In a similar study, Muchtaridi et al. (2017) docked chalcEA and its derivatives in line with Lipinski’s rule to ERα (pdb entry 3ERT) using AutoDock [207,240]. The authors then used 3D pharmacophore modeling (using LigandScount4.1 Advanced validated by actives and decoys taken from DUD-E database) to screen through the derivatives, all of which showed higher docking score than chalcEA [240,241]. Derivatives with a similar dimethylaminoethoxy side chain as OHT, had the best scores but lacked experimental confirmation [240].

In an attempt to target mutated ER, in 2016, Munir et al. used LigandScout for preparation of ERα structure using four pdb structures—1UOM (C381S, C417S and C530S mutations with bound to a tetrahydroisochiolin ligand), 2JFA (M361S, M411S, M483S and M530S with bound raloxifene), 4XI3 (L372S and L536S with bound bazedoxifene) and 1R5K—for the generation of a pharmacophore model based on common features from all three ligand based models and for generating a model based on mutations from all three proteins [242]. ZINC database was screened to find ligands that fit the pharmacophore model using Ligscree Server followed by Lipinksi’s Rule-based filtering to create a ligand database of interest which was docked to the wild-type and the shared mutated protein model with the Patch Dock server [242,243]. The authors were able to obtain 10 hits, out of which three molecules displayed ideal in silico interactions with both wild-type and mutated protein models [242]. Experimental validation should be used for these studies to corroborate these claims, as docking scores are not always reflective of in vivo activity.

Tissue specificity of SERMs for their antagonistic or agonistic profile and E2-independent coactivator binding poses a challenge for using CADD tools for discovering novel modulators. Simulation software such as Simulation plus could be used to explore the tissue specificity problem but it is not an absolute representation of a biological system [40,41]. There is also ambiguity of clear pharmacological differences between agonists and antagonists, which makes it harder to discover specific ligands for ER [244].

#### 3.1.2. Selective Estrogen Receptor Degraders (SERDs)

To address partial agonistic properties of SERMs, purely agonistic SERDs were later introduced. These compounds intend to cause degradation of ER by competitively binding to the EBS, causing conformational changes that target the ER for degradation, thus downregulating ER in the cell [36]. SERDs have been known to destabilize the ER by changing the H12 conformation such that it increases the hydrophobic surface exposure and inhibits ER dimerization.

AstraZeneca developed the most widely known and the only approved SERD–fulvestrant—marketed under the name of Falsodex (Table 3). It has been approved as a second line of treatment for advanced hormone dependent BCa in postmenopausal women. However, there are clinical trial results suggesting that it is effective as a first line treatment in combination therapy in ERα+ and/or PR+ tumors as well [245,246,247]. Fulvestrant inhibits ER dimerization blocking its nuclear localization, even the fulvestrant-bound ER that localizes into the nucleus is transcriptionally inactive [248,249,250,251]. Its structure is similar to that of E2 but with an addition of an alkylsulfinyl group as a side chain to disrupt the H12 resulting in increased surface hydrophobicity. Accelerated degradation of ER due to the instability of fulvestrant bound ER results in downregulation of ER in the cell [246,252]. Fulvestrant has a much higher affinity to the ER than tamoxifen with an IC_50_ of 4.4 nM in inhibition of cell proliferation in ERα+ MCF-7 cells and functions in a dose-dependent manner [245,253,254,255]. Exploring this association may lead to a higher efficacy of the drug. On the other hand, fulvestrant is twice as active on ERβ as on ERα [166]. In some cases, patients have been shown to develop resistance to fulvestrant that has been attributed to *ESR1* or *ERBB2* mutations [247]. Moreover, it cannot be administered orally due to poor aqueous solubility and bioavailability and thus has to be delivered intramuscularly through injections which limits the dosage [250,256].

**Figure 8:**
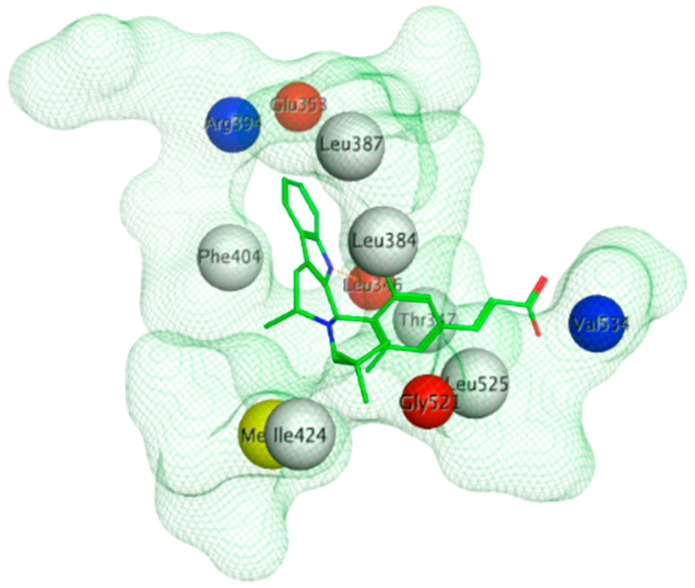
Site view of AZD9496 (green) in complex with ERα-EBS (pdb: 5ACC). The indole ring of AZD9496 forms a hydrogen bond (dashed, orange) with Leu346.

The next generation of orally administered SERDs with high bioavailability are AZD9496, elacestrant, LSZ102 and GDC-0927, as also presented in Table 3. Of those, AZD9496 was identified using iterative SAR by Weir et al. and has been in clinical trials since 2014. The compound exhibits antiproliferative IC_50_ of 0.03 nM on MCF-7 cells, and is highly selective towards ER [257]. We used MOE to inspect the interaction between AZD9496 and ERα as shown in Figure 8 [42]. In AZD9496 bound complex with ER, the nitrogen atom in the indole ring of AZD9496 forms a strong hydrogen bond with Leu346 residue [257]. The H12 region of ER is destabilized due to acid–acid overlap between the side chain of the molecule and Asp351 which has been attributed to degradation of the protein [258,259]. Phase I studies of AZD9496 in advanced ERα+/HER2- BCa patients found the drug to be tolerable and safe [260]. Other studies demonstrated AZD9496 to be comparable to fulvestrant in cell growth inhibition as well as cross-resistant to fulvestrant [261].

Elacestrant/RAD1901 is a SERM/SERD hybrid substance developed by Hattersley et al. for Radius Pharmaceuticals in 2015, and is currently in Phase III trial aiming to compare its safety and efficacy to the standard endocrine therapy in combination with fulvestrant or AI in advanced ERα+/HER- setting [262]. It works in a dose-dependent manner in degradation of ER and exhibits agonistic SERM like activities at doses that do not induce ER degradation [263,264]. It resulted in growth inhibition of MCF-7 cells and *ESR1* mutated xenograft models (including fulvestrant and tamoxifen resistant ones) [263,265,266].

**Figure 9:**
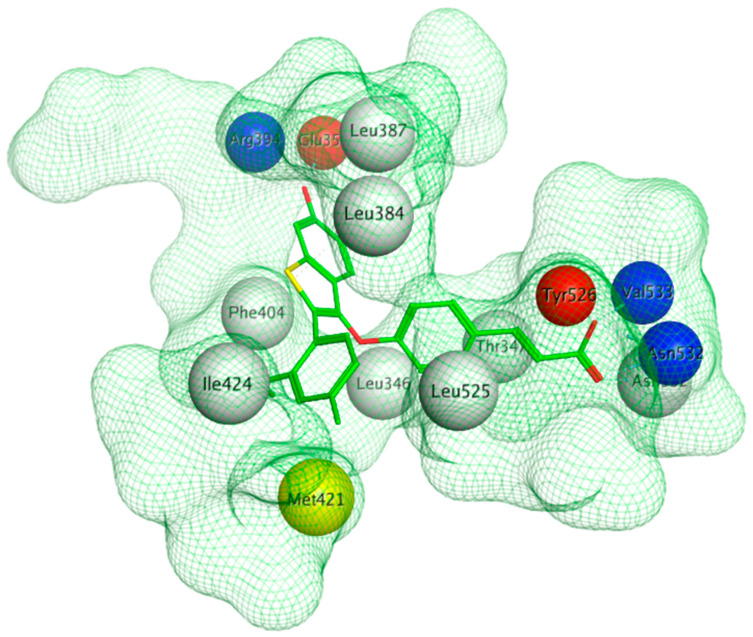
Site view of LSZ102 (green) in complex with ERα-EBS (pdb: 6B0F).

Tria et al. synthesized LSZ102 by combining a benzothiophene core such as raloxifene/arzoxifene and a carboxylic side chain from GW-7604, which is a precursor of a previously undeveloped drug etacstil/GW-5638 by Novartis Pharmaceuticals [253]. It is currently in Phase I trials aiming to determine the safety and tolerability as a monotherapeutic agent or in combination with LEE011 (a CDK4/6 inhibitor) or BYL719 (a PI3K inhibitor) in advanced ERα+ BCa [267]. Using MOE, we can see that the phenolic hydroxyl group of LSZ102 forms hydrogen bonds with Arg394 and Glu353 residues of ER, while the rest of the core is involved in hydrophobic interactions with the protein (Figure 9) [42,253]. The van der Waal forces due to the difluoroethyl group on the compound result in a conformational change of the Phe425 side chain increasing the size of the cavity [253]. The bulky side chains of both AZD9496 and LSZ102 cause a conformational change in H12 that ultimately destabilizes the ER thereby inducing degradation [253]. The compound displayed an IC_50_ of 6 nM in ER transcription and 0.2 nM in ER degradation in ERα+ MCF-7 cells. With an IC_50_ of 1.7 nM for inhibition of proliferation of MCF-7 cells, it is significantly more potent than fulvestrant and toremifene [253].

**Figure 10:**
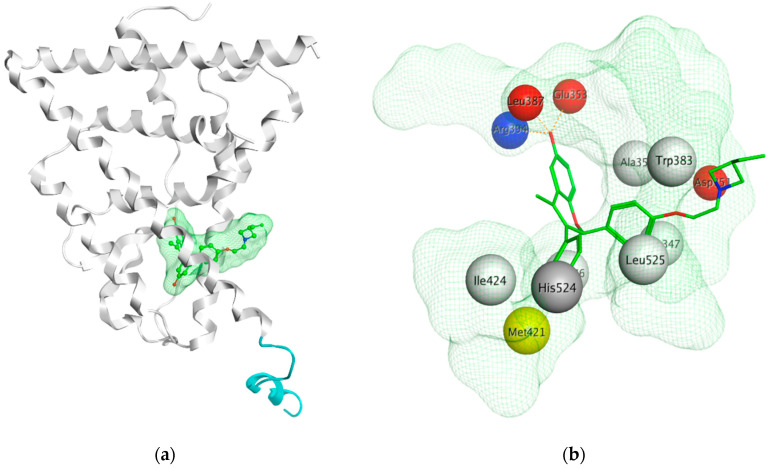
(**a**) GDC-0927 (green) in complex with ERα (white) with H12 in cyan; and (**b**) site view of GDC-0927 (green) in complex with ERα-EBS (PDB: 6PFM). The dashed lines (orange) represent the hydrogen bonds formed between GDC-0927 and Arg394, Glu353, Leu387, His524 and Asp351.

To target tamoxifen resistant cells, Kahraman et al. synthesized GDC-0927 (IC_50_ 0.1 nM) for Seragon Pharmaceuticals in collaboration with Genetech Inc. This SERD causes 97% degradation of ERα and is highly active in tam-resistant MCF-7 xenografts [268]. In Phase I trials, it was determined that the compound was safe and tolerable in metastatic ERα+/HER- BCa in post-menopausal women including patients harboring *ESR1* mutations [269]. The structure of GDC-0927 in complex with ERα has been resolved but the H12 seems disordered in its packing to the rest of the ER as seen in Figure 10a using MOE package [42,270]. The authors have not made it clear if this is due to the nature of the compound or the quality of the crystal itself. Apart from that, GDC-0927 forms hydrogen bonds on one side with Glu353, Arg394 and Leu387 and on the other side with His524 along with a hydrogen bond interaction between Asp351 and the azetidine nitrogen in the side chain (Figure 10b) (pdb entry 6PFM) [42,270].

To summarize this section, it is necessary to note, that some compounds exhibit dual SERMs and SERDs profiles that make it difficult to clearly differentiate their mode of action. Furthermore, since such SERD molecules bind to the exact same site as modulators, it faces the same challenges for using CADD tools for novel discoveries.

#### 3.1.3. PROTAC Ligands

Another marked method for ER degradation is the recruitment of proteolytic E3 Ubiquitin ligase using Proteolysis-targeting chimeras (PROTACs) technology. The use of PROTAC approach would remove any ambiguity that arises from structurally similar SERMs and SERDs. In particular, PROTAC ligands represent essentially two linked active molecules—one binding to the protein to be degraded (in this case, ERα) called the core, and the other binding to E3 ubiquitin ligase (called a degron). General structure of a PROTAC includes an R1 motif that binds to the protein to be degraded; R2, which is the linker; and R3 is the degron. There has been a newfound interest in repurposing already established small molecule inhibitors specific to the targeted protein for PROTACs (Table 4).

The first PROTAC degrader of ER was reported by Sakamoto et al. (2003) who used E2 linked to IκBα phosphopeptide degron (termed as Protac-2) to target ER for mediated degradation with SCFβ-TRCP ubiquitin ligase [271]. Protac-2 treatment resulted in dose dependent ER ubiquitination and degradation by yeast 26S proteasomes in vitro but not in vivo due to technical difficulties posed by ER reporter protein expressing cells [271]. In 2008, they developed another compound, PROTAC-B, which was an E2-VHL (von Hippel–Lindau) ligase complex with a HIF-1α pentapeptide degron which inhibited proliferation of MCF-7 and T47D cell (IC_50_ values of 50 μM and 16 μM, respectively, at 72 h), but had no effects on ERα- SKBr3 cells [272]. PROTAC-B results in ERα degradation through proteasomes since its activity can be blocked by MG132, while adding E2 in a system with PROTAC-B decreases its ability for ERα degradation [272]. The compound causes G1 cell arrest in MCF-7 and T47D cells as it downregulates cyclin D1 and PR expression [272].

**Figure 11:**
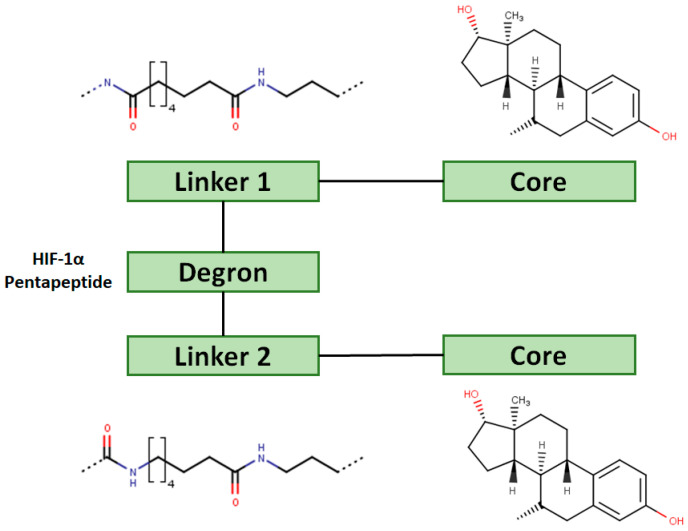
A double headed PROTAC with two E2s to bind to proteins with a common degron developed by Kim et al. [274].

For optimal linker position on E2, Kim et al., in 2010, tested E2 linked to an E3 recognition residue degron at various sites. C-terminal protected PROTAC, protected penta-peptides and C7α linked compounds provided more substantial degradation. Competitive ligand binding assay suggested that “Compound 24” which was based on C7α linkage at the E2 had a higher affinity than tamoxifen [273]. In the same year, they synthesized a novel two headed PROTAC molecules with two E2 molecules linked at the previously reported C7α linkage site with HIF-1α pentapeptide (VHL ligands) degron causing proteasomal ERα degradation (Figure 11) [274]. Competitive ligand-binding affinity assay established that the two headed PROTAC was three times more active and resulted in higher than fivefold ERα degradation, but displayed poor solubility with respect to its monomeric counterparts with either the N terminal or C terminal linkages to the VHL ligand [274].

In 2011, Itoh et al. synthesized a new type of PROTAC ligands termed SNIPERs (Specific and Nongenetic IAPs-dependent Protein ERasers) intended for nuclear receptors—retinoic acid receptors, AR and ERα [275]. Known ER agonist E1 was used for targeting ERα linked to cellular inhibitor of apoptosis protein 1 (cIAP1) ligand degron to create “Compound 11” (Table 4) [275]. Western blots concluded that “Compound 11” downregulated ERα in MCF-7 cells [275]. In continuation of this study, Okuhira et al. developed SNIPERs for ERα degradation and induction of BCa cell death via necrosis in 2013 [276]. The authors used OHT in conjugation with bestatin, a cIAP1 ligand to synthesize three compounds with different linker lengths with similar activity at 6 h in MCF-7 cells but SNIPER(ER)-3 was found to be more active at 24 h [276]. At 3 µM, the compound resulted in increase of ERα levels attributed to OHT’s antagonistic SERD-like activity, but, at 30 µM, the compound downregulated ERα and cIAP1 levels in both MCF-7 and T47D cells, indicating that the compound caused ubiquitylation and further degradation of both ERα and cIAP1 [276]. qRT-PCR on SNIPER(ER)-3 treated cells indicated that the compound successfully inhibited the upregulation in pS2 mRNA stimulated by E2 [276]. The compound induced cell death via necrosis in MCF-7 cells but not in U2OS and HeLa cells or MG132 treated MCF-7 cells indicating that the necrosis was linked to ERα degradation and production of reactive oxygen species [276].

In 2018, an N-terminal aspartic acid cross linking (TD) strategy for stabilizing the peptides and achieving increased cell-permeability was developed by Jiang et al. [277]. They synthesized three peptide-based PROTACs (TD-PROTACs) to target ERα with a TD peptidomimetic ER modulator (TD-PERM) bound to a VHL ligand degron [277]. The peptide TD-PROTAC having a 6-aminohexanoic acid linker showed activity immunofluorescence and flow cytometry assays. Immunoblotting was able to establish dose dependent ERα degradation in T47D cells with DC_50_ < 20 µM and showed reduced activity against PR and Vitamin D receptor [277]. The peptide caused proteasome mediated degradation of ERα through ubiquitylation and was found to be active on MCF-7 and T47D cells but not MDA-MB-231 and HEK 293T cells [277]. TD-PROTAC inhibited receptor signaling as supported by a decrease in pS2 mRNA measured using qPCR and induced S-phase arrest in T47D cells [277]. Nude mice xenografted with MCF-7 cells and injected with 10 mg/kg TD-PROTAC resulted in 75% reduction in tumor volume in 42 days similar to 4 mg/kg tamoxifen in comparison with the control untreated tumor [277].

Sharma et al. used an ERα ligand based on previously developed a SERD with 2–3 times more affinity towards ERα than E2 to increase PROTAC activity [278]. The ER ligands were linked to various E3 ubiquitin ligands with either lipophilic amino acid, monocyclic, bicyclic or tricyclic motifs [278]. The lipophilic amino acid Boc-Trp motif resulted in significant anti-proliferative effect (IC_50_ 16 nM) and ERα downregulation with IC_50_ value of 0.5 nM [278]. The monocyclic trifluoromethyl cyclohexane motif containing PROTAC could reach an antiproliferative IC_50_ value of 0.5 nM and ERα downregulation IC_50_ value of 2 nM while, the C3-linked adamantane motif tested could reach an antiproliferative IC_50_ value of 9 nM and ERα downregulation IC_50_ value of 3 nM [278]. The compounds could downregulate ERα mediated transcription of PR, pS2, and GREB1 mRNA and were active even at higher concentrations [278].

In 2019, Hu et al. developed a PROTAC, ERD-308, using N,N diethylamino raloxifene analog with a cereblon or a VHL ligand for degradation of ER [279]. Initial testing by Western blots confirmed that VHL ligand was more effective as ER degrader resulting in ERD-148 with an IC_50_ value of 196 ± 6 nM [279]. Various linkers were tested for optimizing ERD-148’s activity leading to linker containing a polyethylene glycol unit, achieving >95% inhibition at 5 nM concentration in ERα+ T47D BCa cells and >80% degradation at 30 nM in MCF-7 cells [279]. The compound had an IC_50_ value of 0.77 nM in MCF-7 cells cell proliferation assay with an I_max_ value higher than both fulvestrant, raloxifene and elacestrant at 57.5% which for further evaluated by crystal violet staining and had no effect on ER- MDA-MB-231 cells [279]. Analysis of ERD-308 on MCF-7 cells using qRT-PCR further found that the compound successfully downregulated levels of PR and GREB1 mRNA [279]. The group then synthesized compounds based on ERD-148 by substituting N,N diethylamino raloxifene domain with tamoxifen, OHT, bazedoxifene or lasofoxifene but ERD-148 was found to be the most potent among all [279]. Although we could not find any example of this, CADD tools can assist the development of new PROTACS. VS for various cores and degrons along with docking simulations with different cores, linkers and degrons could aide initial testing of these molecules.

#### 3.1.4. LBD-Directed Covalent Inhibitor

**Figure 12:**
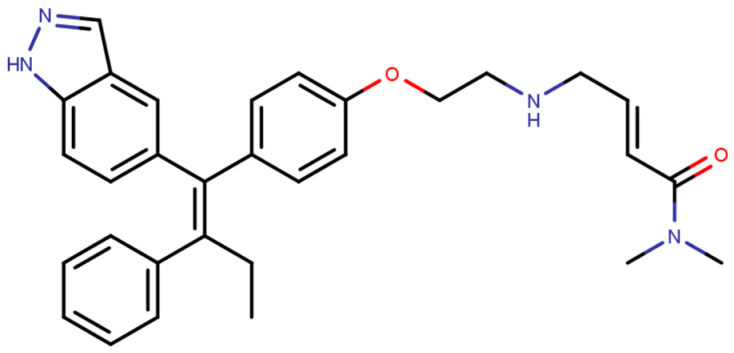
Structure of LBD-directed covalent inhibitor, H3B-5942.

In the quest for a class of drugs with improved potency and ability to overcome development of resistance to EBS directed drugs, Selective ER covalent antagonists (SERCAs) were also developed. SERCAs inhibit ERα activity by covalently binding to the ER-LBD. Recently, Puyang et al. discovered H3B-5942 (Figure 12), an orally available SERCA that was found to be safe and tolerable in ERα+/HER−BCa patients in Phase I clinical trials and is currently in Phase II trials [280].

The compound inhibits ERα mediated genes *GREB1* and *TFF1* on wild type ERα and its mutant forms, Y537S, Y537N, Y537C and D538G [281], while displaying no agnostic activities on endometrial cells as SERMs and SERDs do [281]. This suggests that the mechanism of action of SERCA may be different from traditional EBS directed therapies.

**Figure 13:**
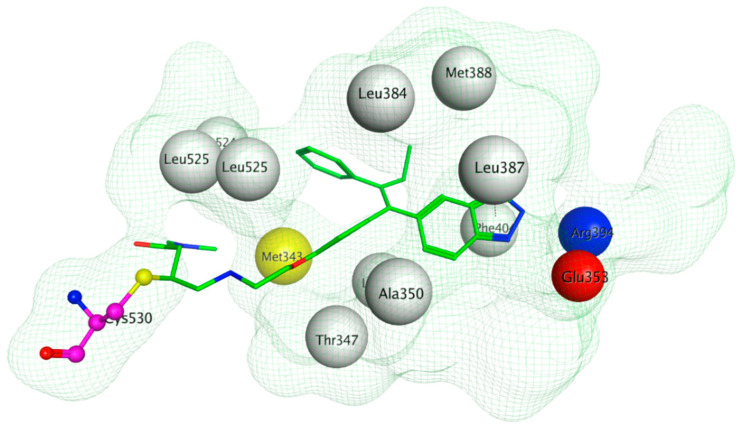
Site view of H3B-5942 (green) in complex with ERα-EBS. It forms a covalent bond with the residue Cys530 shown in pink (pdb: 6CHW).

The crystal structure visualized using MOE software demonstrated that H3B-5942 forms covalent bond with Cys530 that is located at the C-terminal end of H11 and directly involved in AF2 pocket formation (Figure 13) [42,281]. The rest of the H3B-5942 occupies the EBS cavity in the ER-LBD [281]. Combination treatment with CDK4/6 or mTOR inhibitors has shown improved efficacy [281]. However, H3B-5942 could result in Cys530 mutation resulting in decreased efficacy of the drug [281]. The effects of this compound on all known forms of ERα mutants are not yet known and may result in resistance via non-genomic signaling as seen in the case of tamoxifen resistance [282].

As the previous review sections illustrate, EBS directed drugs for years have been the norm in targeting ERα (Table 5). There are many promising inhibitors currently under clinical trials that are more potent and have less unwanted side effects compared to tamoxifen and fulvestrant. However, these inhibitors have not yet overcome the high frequency of resistance development in BCa cells. Moreover, some mutant forms of ER have been shown to recruit co-regulators and thus be transcriptionally active even in the absence of hormones. Thus, there is still an unmet need to consider targeting other sites on ERα to overcome the limitations of EBS-directed strategies.

### 3.2. AF2-Directed Inhibition

**Figure 14:**
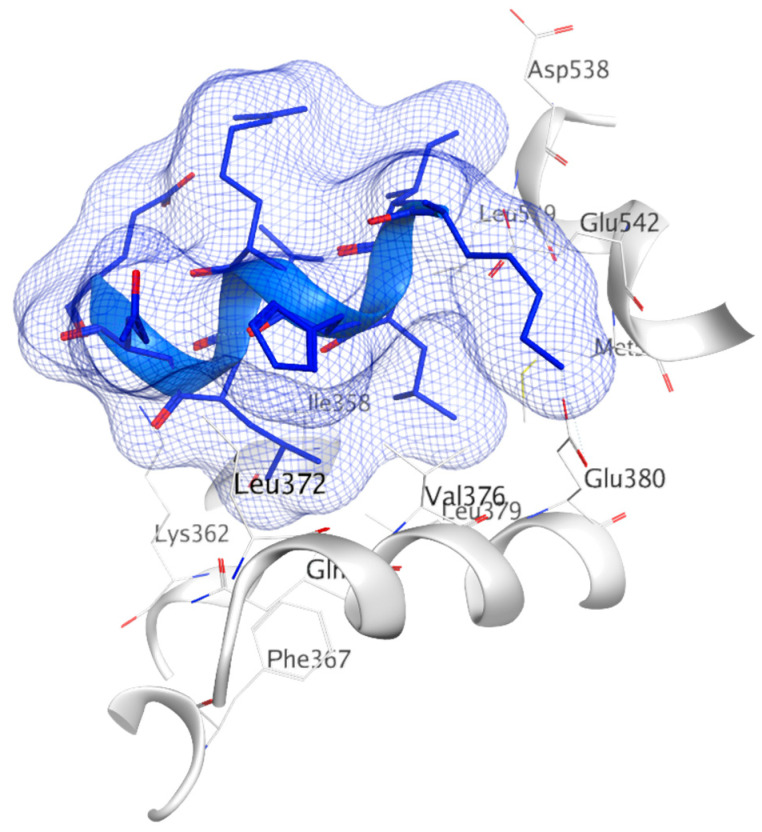
A coactivator peptide LxxLL motif of the coactivator Steroid receptor Coactivator (SRC-1) (blue) docked to AF-2 site on the ER-LBD (white) (pdb: 3UUD).

BCa cells develop resistance to traditional HT over time, and, therefore, targeting alternative sites on ERα may provide effective treatment options. One such target pocket that has been of particular interest is the AF2 functionality of the ER-LBD (Figure 14). It has been demonstrated that coactivator p300 can bind to the AF2 pocket even in the absence of E2, by using the notch signaling pathway [283]. Moreover, certain ERα mutants have also been shown to be transcriptionally active in the absence of hormone, and therefore such mutants are unresponsive to traditional AI or SERM/SERD therapies [112,113,115,284]. Thus, blocking AF2-coactivator interaction could represent a very practical approach to prevent ERα transcription in drug-resistant BCa. In recent years, molecular modeling has advanced tremendously offering an indispensable tool-box for evaluation of small molecule binding sites on surfaces of protein targets. Besides the EBS on ER-LBD, the AF2 site has been visualized by X-ray structures of ER-LBD. The site can be found using MOE SiteFinder, as shown in Figure 14, where small molecule inhibitor can substitute co-activator [42].

The AF2 cavity is a well-defined hydrophobic pocket made up of Leu354, Val355, Met357, Ile358, Asn359, Lys362, Phe367, Val368, Leu370, Thr371, Leu372, His373, Gln375, Val376, Leu179, Trp383, Leu539, Glu542, Met243 and Ala546 residues. As discussed above, many co-regulators containing a hydrophobic LXXLL motif similar to H12 interact with this pocket [285].

Recently, Raj et al. discovered ERX-11, which is a highly potent, non-toxic, and bioavailable compound (Table 6) that inhibits ERα via AF-2 mediated mechanism of action [284]. It exhibits IC_50_ values ranging from 250 to 500 nM for growth inhibition of ERα + cells and inhibits growth of both wild type and mutant forms of ERα with comparable potency [284]. ERX-11 reduces tumor volume by 73% in MCF-7-PRLPS1 xenografts. The compound also demonstrates significant activity in Tam-resistant and letrozole-resistant cell lines [284,286]. ERX-11 in combination with a palbociclib (CDK4/6 inhibitor) was found to be more potent in tamoxifen and letrozole resistant cells than either treatment alone [287,288]. Lead optimization based on ERX-11 yielded four compounds with nanomolar activity against ERα, which are currently being validated in preclinical phase [287]. Essentially, the two iso-butane groups of ERX-11 mimic the LXXLL motif of the co-activator and fill up the volumes of leucine side chains of the LXXLL motif [284]. This compound is one of the most promising compounds developed so far that targets an alternative site on ERα.

Many previous attempts have also been made for targeting this pocket. Thus, in 2004, Katzenellenbogen et al. proposed this site as an alternative strategy to target ERα using small-molecules by showing the inhibitory effects of compounds with pyrimidine core on binding of E2 activated ERα with a labeled SRC-1 Box II peptide using an FP assay [289]. Their best compound (termed as Compound 12A in the paper), displayed Ki values of 29 µM which made it unfavorable for further advancement in biological assays or in vivo studies [289]. In 2007, they synthesized bicycle [2.2.2] octanes to target the ER-AF2 site by essentially *de novo* mimicking the two Leu residues in the binding motifs of SRC [290]. However, the new compounds displayed much lower potency than the previous pyrimidine core with Ki values ranging 7–40 µM compared to 0.91 µM for the previously reported “Compound 12A” and 0.19 µM for SRC-1 Box II peptide using time-resolved fluorescence energy transfer (TR-FRET) assay [290]. The best hit, named ”Compound 18” (Table 6), demonstrated IC_50_ of 17.1 µM [290]. To improve potency of previously reported compounds, in 2008, the same group continued with SAR studies around the pyrimidine core and reported molecules with improved Ki of 2–3 μM in a TR-FRET assay, with the best molecule, termed “Compound 11c”, demonstrating Ki value of 1.7 μM [291]. In the same year, amphipathic benzenes, synthesized by mimicking the Leu-rich SRC, demonstrated improved solubility compared to pyrimidines, with most of developed inhibitors (Compounds 3c–e in Table 6) exhibited Ki values of 1.7–2.1 μM in TR-FRET assay and low micromolar Ki values in both reporter gene and mammalian two-hybrid (M2H) assay in HEC-1 cells (Table 6) [292]. In 2011, the group used HTS to identify novel hits with IC_50_ values in 2.3–5 μM range with their best hit “Compound 1g” shown in Table 6 [293]. The authors further studied SAR in the series based on the docking poses generated by Glide and subjected to MD simulation for water displacement and identified that hydrophobic Ile689, Leu690, Leu693 and Leu694 residues in SRC are important for binding to the ERα [293]. The findings suggested that more focus should be put on larger compounds to target his site [293].

In 2007, Becerril and Hamilton also tried to mimic leucine side chains of coactivator peptide to synthesize multiple chemicals out of which “Compound 7” was able to achieve 4.2 μM Ki value in an FP assay [294]. Researchers from Wyeth Pharmaceuticals performed a study that combined high-throughput M2H assays with virtual screening to identify novel co-activator binding inhibitors [295]. They reported guanylhydrazone compound ERI-05 (IC_50_ = 5.5 μM) that blocks the interactions of Gal4 DBD/ERα LBD fusion and SRC-1, SRC-3 or SRC-3/VP16 21 fusion in a M2H assay performed in COS7 cells [295]. Although ERI-05 reduced the expression of the ERα regulated gene *pS2* in MCF-7 cell line at 20 μM, it turned out to be toxic at higher concentrations [295]. Subsequently, Katzenellenbogen et al. developed a series of guanylhydrazone-based inhibitors based on previously reported activity of the said core with reported inhibition of ERα transactivation in MCF-7 cell line with some showing improved potency over ERI-05 [296]. The compounds produced displayed lower μM IC_50_ values mostly within 1–8 μM for reporter gene assays and around 2.6–12 μM for M2H assays [296]. Compounds 20, 22 and 29 were some of the best hits, as seen in Table 6 [296]. Since these compounds react covalently with nucleophilic residues in ERα, they could not be explored further [296]. Additionally, peptide inhibitors (designed based on LxxLL motif) were reported to inhibit the interaction of ER with co-activators [296]. However, their application is limited by poor permeability and lack of specificity [296].

In 2015, Singh et al. used CADD in the discovery of an ERα-AF2-directed compound, VPC-16230, through in silico screening of compounds from the ZINC15 database with Glide (Schrodinger Software) and eHiTs docking [43,297,298]. Consensus scoring based on various factors such as- docking score, pKi values, RMSD values for voting along with visual inspection and in vitro testing were used for discovering potential hits [297]. VPC-16230 could significantly decrease mRNA expression mediated by E2, inhibit proliferation of MCF-7 cells at an IC_50_ value of 7.8 μM, and Tam-resistant TamR3 cells and TamR6 cells at IC_50_s of 3.4 μM and 6.3 μM respectively, with no detected suppression of ERα- MDA-MB-453 and HeLa cells [297,299]. Molecular similarity search using VPC-16230 as a template found another more potent compound VPC-16464 with an IC_50_ value of 2.7 µM [300]. Lead optimization on the basis of VPC-16464 led to VPC-16606, which inhibits ERα co-regulator binding with an IC_50_ of 0.3 μM [300]. It downregulates ERα-dependent mRNA expression and was found to be ERα selective over other SHRs (PR, GR and AR) [300].

### 3.3. ER DBD-Directed Inhibition

**Figure 15:**
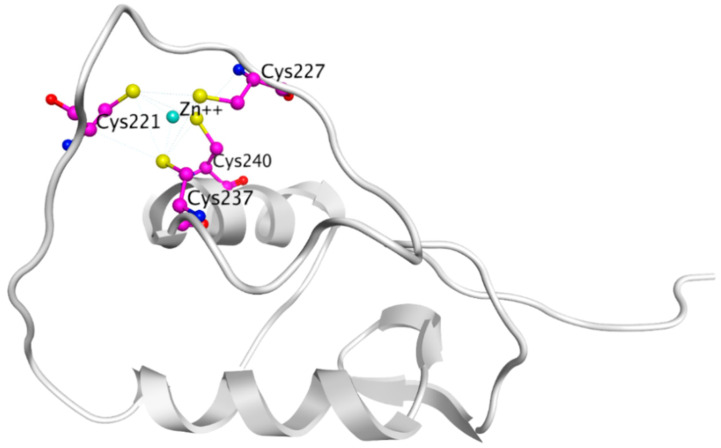
The second zinc finger on the ER-DBD (white). Cys residues are shown in pink (pdb: 1hcq).

Another strategy to inhibit ERα could be to target the ER-DBD functionality, which represents the actual active site of all nuclear receptors. The two regions for small molecular inhibition are P-box or the D-box [79,80]. Compounds causing functional disruption of either boxes may lead to a novel class of ER-directed drugs. Employing the MOE visualization package, the second zinc finger can be seen in Figure 15 [42]. This zinc finger forms the DBD/DBD dimer interface in the DBD and has thus been the target for many inhibitors. The following molecules have been found to be ER-DBD-directed (Table 7).

In a paper published in 2003, Wang et al. documented that 2,2′- dithiobisbenzamidine (DIBA) and benzisothiazolone (BITA) treatment of MCF-7, T-47D and ZR-75 cells resulted in the inhibition of E2 driven proliferation of the cells in a dose-dependent manner (Table 7) [301]. In an extended study on the compound DIBA, in 2006, the same authors found that the small molecule was also able to inhibit tumor proliferation, decrease tumor mass in a dose dependent manner at a high dose of 30 mg/kg and restore tamoxifen sensitivity, which has been attributed to modification induced by DIBA on ER [302]. DIBA was also found to selectively inhibit ER activity over other SHRs [301,302,303]. Inhibitory effects of electrophiles such as DIBA and BITA on Zinc finger activity in tamoxifen-resistant BCa cells have been shown for both ligand independent and dependent signaling (Table 7) [301]. The mechanism of action of DIBA and BITA is to disrupt the labile second ER zinc finger structure in ER by chelation of zinc using weak electrophiles which had been previously studied and documented by Maynard and Covell in 2001 [304].

In 2007, Kim et al. demonstrated that 15-Deoxy-Delta-12,14-prostaglandin J2 (15d-PGJ2), an anti-inflammatory prostaglandin, can inhibit ERα (Table 7) [305,306]. At low concentrations of 0.5–2.5 µM, 15d-PGJ2 suppressed both E2 dependent and independent proliferation of ERα+ MCF-7 cells, but had little effect on ERα- MDA-MB-231 cells [306]. However, at higher concentrations (≥5 µM), it inhibited proliferation in both cell lines [306]. The inhibition of proliferation was attributed to direct covalent modifications of Cys227 and Cys240 in the C-terminal Zinc fingers in the ERα DBD by 15d-PGJ2 and thus disruption of transcription of ER dependent genes [306,307].

A similarly active 8-benzylsulfanylmethyl-1,3-dimethyl-3,7-dihydropurine-2,6-dione (TPBM) was discovered through HTS using in vitro Fluorescence Anistropy Assay (FAA) microplate assay by Shapiro et al. in 2008 (Table 7) [308]. TPBM was found to inhibit ERα DBD binding to fluorescently labeled ERE at an IC_50_ value of 3 µM, E2 dependent growth in BG-1 cells at an IC_50_ value of 5 µM, and E2-ERα mediated gene expression at an IC_50_ value of 9 μM [308]. The compounds were ineffective for hormone independent growth of BG-1 cells and was non-toxic to ERα-MDA MD-231 cells [308]. It was further demonstrated that the inhibitor is noncompetitive and selective toward ER over other SHRs (AR, PR and GR) [308]. The mechanism of action of TPBM is still unknown, but it was shown to be different from other DBD targeting molecules such as DIBA, a Zinc finger-chelator [308,309].

Inhibitory effects of Anacardic acid (AA) on ERα was found by Schultz et al. in 2010 using an electrophoretic mobility shift assay (Table 7) [310]. Molecular modeling with surflex docking suggested that AA inhibits ER-DBD/ERE binding [310,311]. In subsequent studies, Li et al. (2015) and Zhao et al. (2018) found that AA downregulates CDK-4 and induces inhibition of Hsp90 causing cell cycle arrest in MDA-MB-231 cells [312,313]. AA exerts noncompetitive inhibitory effects on proliferation of E2 dependent and independent cancer cells and also tamoxifen-resistant cancer cells [310]. Selectivity of AA towards ER over other SHRs has not been assessed. AA inhibited both ERα and ERβ binding to ERE with IC_50_ values of 13.5, 5.3, and 14 µM in ERα+ cell lines MCF-7, LCC9 and LY2, respectively, and 29 and 39 µM in ERα-/ERβ+ cell lines MCF-10A and MDA-MB-231, respectively [309,310,314].

Thus far, no group has tried using CADD for DBD-directed inhibition as there has not been a widespread interest in developing these inhibitors. CADD tools could prove useful in discovering selective inhibitors for ER.

### 3.4. Dimer Inducers

**Figure 16:**
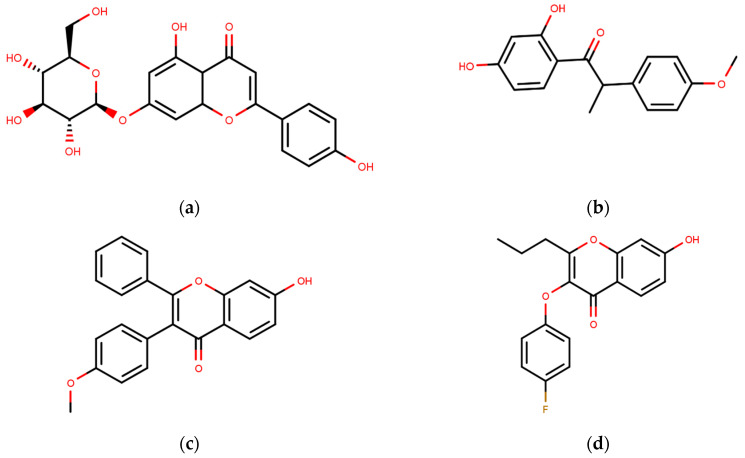
(**a**) Cosmosiin; (**b**) angolesin; (**c**) Compound 9; and (**d**) Compound 10.

ER has the ability to form both heterodimer and homodimers with ERα homodimers promoting cell growth and ERβ homodimers and ERα/ERβ heterodimers inhibiting the same. Inducing ERβ homodimers and ERα/ERβ heterodimers and decreasing ERα/ERα homodimer formation using small molecules would therefore represent a viable strategy for BCa inhibition [315,316]. In an intriguing development, Xu and group developed a bioluminescence resonance energy transfer (BRET) assay to distinguish compounds on the basis of their ability to form different dimers and found that phytoestrogens, cosmosiin and angolesin, transcriptionally activate ERα/ERβ heterodimers at 1 and 10 µM, respectively (Figure 16a,b) [315,316,317]. They inhibit proliferation and migration of cells expressing both ERα and ERβ but not of ER- or ERα-/ERβ+ cell lines [317]. Dimer formation was quantified using the BRET assay [315,316]. Elaborating on their previous work, the same group in 2018 used the same assay to screen flavonoids that displayed activity in reporter-based assays in T47D-KBluc reporter cell line for their dimerization selection [315,318]. Seven compounds (including cosmosiin) were then used to build a pharmacophore model using GALAHAD software [318,319]. The best pharmacophore model was chosen on the basis of Pareto ranking (pharmacophore-based similarity), energy and steric score providing a model with seven essential features including three acceptor atoms, one donor atom and three hydrophobic centers on phenyl and benzopyran rings of cosmosiin [318]. The models were then converted to 3D search query using UNITY-3D (SYBYL software) and used for a non-restrictive, flexible screening through Maybridge and Chembridge databases (taken from ZINC database) producing a list of hits that fit the model [318]. Qfit and SYBYL were used to rank compounds that fit all seven features of the pharmacophore model (167 compounds), purchasing the top 22 hits [318,320]. T47D-KBluc reporter based assay was again used to confirm their activity and BRET assay in HEK293 cells for their preferences giving four compounds (Compounds 4, 6, 9 and 10, as named in the paper) that selectively induce ERα/ERβ heterodimers at 1 M [318]. “Compound 4” and “Compound 6” displayed high IC_50_ values for both ERα and ERβ, while IC_50_ values of “Compound 9” were 1.4 and 2.0 µM and “Compound 10” were 1.9 and 3.2 µM for ERα and ERβ, respectively (Figure 16c,d) [318].

### 3.5. Targeting DBD/LBD Interface

**Figure 17:**
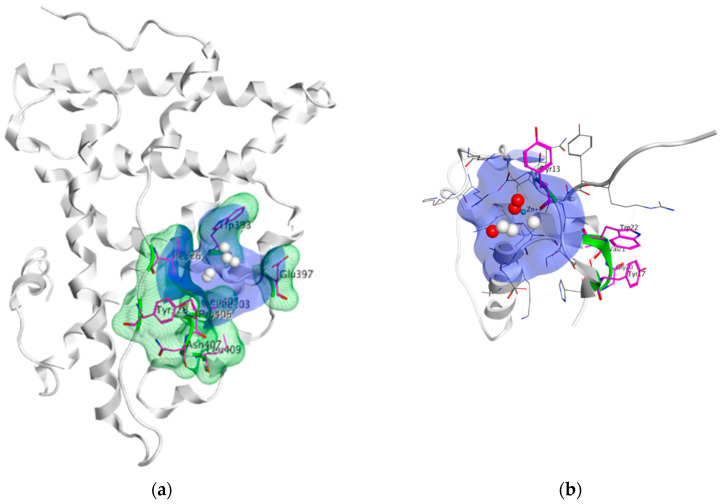
(**a**) The important residues (green surface, pink atoms) of ER-LBD that interact with ER-DBD. The identified binding site is shown in blue. (**b**) The important residues (green ribbon, pink atoms) of ER-DBD interacting with ER-LBD.

The LBD/DBD interaction surface is essential for ER transcription; thus, disruption of the interdomain crosstalk has been proposed as another strategy to inhibit ER function. Ile326, Tyr 328, Trp393, Glu397, Leu403, Pro406, Asn407 and Leu409 of the ER-LBD make up the interface that interacts with residues Tyr 191, Trp200, Tyr195, Val199 and Gly198 of ER-DBD [321]. Wells et al. (2007) explored the surface using hydroxyl radical-based protein footprinting, computational modeling and site-directed mutagenesis [321]. Site directed mutations in the implicated ER-LBD interface (I326A, Y328A, P406A, and L409A, see Figure 17a) have been shown to inhibit E2 dependent transactivation without hampering the ability of the ER to bind to E2 and the coactivator proteins [321]. More importantly, MOE SiteFinder has detected small molecule binding sites on ER-LBD and ER-DBD (shown in Figure 17a,b, respectively) [42]. The site on ER-LBD seems more functionally prominent. To design small molecule inhibitors for direct disruption of the LBD/DBD interaction, more functional and structural knowledge is required.

### 3.6. Targeting F-Domain

**Figure 18:**
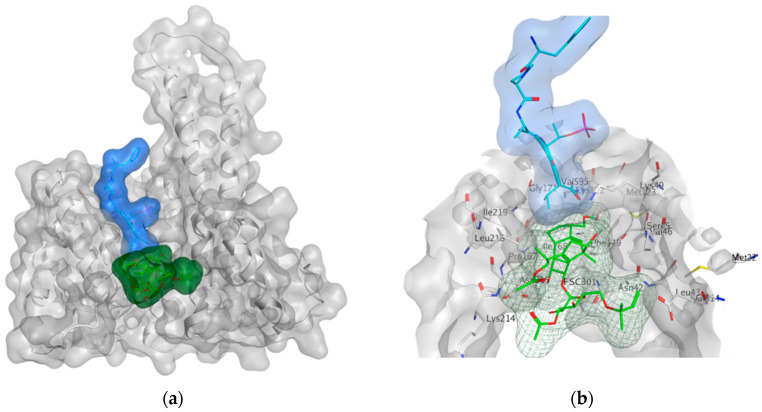
(**a**) Fusicoccin (green) stabilizing ERα (Blue) and 14-3-3 (grey) complex (pdb: 4JDD); and (**b**) site view of fusicoccin (green) interaction with ERα (blue) and 14-3-3 (grey) (pdb: 4JDD).

The F-domain interaction with regulatory proteins such as 14-3-3 family of proteins reduces E2 independent transcription and inhibits ER dimerization [99]. Enhancing this interaction increases the 14-3-3 inhibition effects on the ER dimerization, which should result in a great strategy to inhibit ERα [99]. Thus, in 2013, De Vries–van Leeuwen demonstrated that the small molecule of fusicoccin stabilizes the binding of F-domain to 14-3-3 protein, as shown in Figure 18 [99]. Fusicoccin acts as a “molecular glue” between the F-domain and 14-3-3 interaction. The binding site of the molecule is the mode-III site consisting of Asp215, Lys49, Lys122, Leu218, Ser45, Met22, Glu14, Leu43, Asn42, Phe119, Ile169 and Lys214 residues, as shown in Figure 18, with the help of MOE visualization package, where fusicoccin forms H-bonds with Asp215, Lys49 and Lys122 of the 14-3-3 protein [42,99].

Recently, Sijbesma et al. attempted to target this interaction using site-directed small fragment-based screening [100]. The phosphopeptide (mimicking ERα F-domain) interaction with 14-3-3 were stabilized by about 40 times using disulfide fragment tethering of the native Cys38 and the two mutated Cys42 and Cys45 [100].

### 3.7. Targeting Binding Function-3 (BF3)

**Figure 19:**
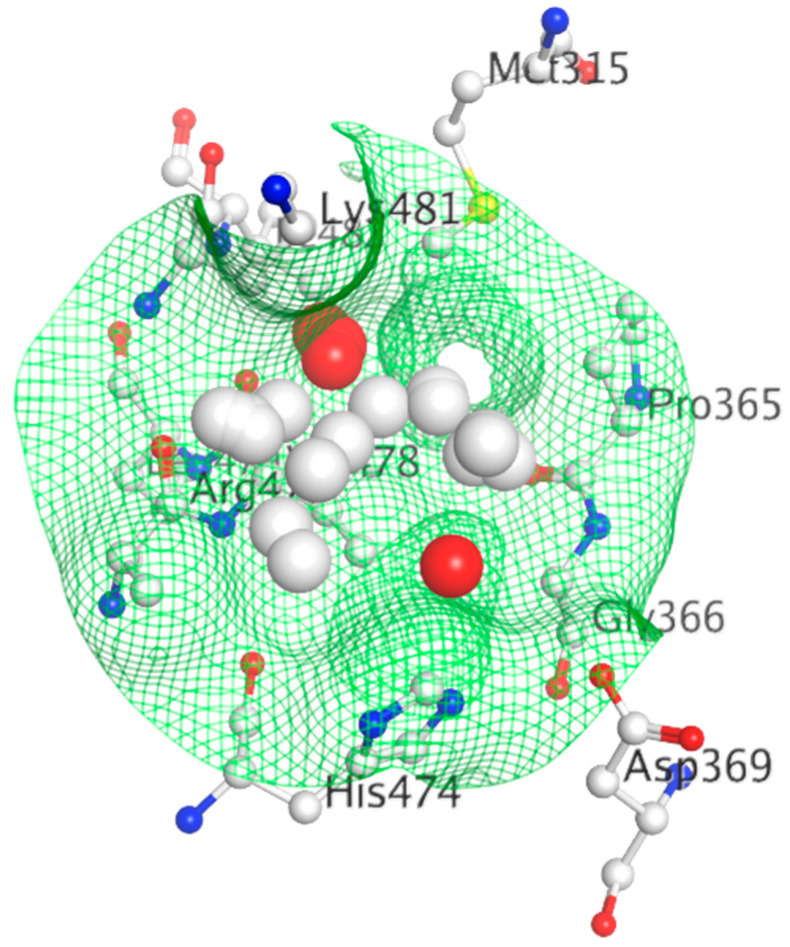
Location of potential BF3 site on ER-LBD fold (pdb: 3UUD).

Based on the homology between ER and AR, a potential small molecule binding BF3 site has been proposed using MOE software, although no experimental evidence of the BF3 functionality has been provided yet for ER, as it has been previously established for AR [42,322,323,324,325]. Due to functional similarity between the SHRs, the dynamic nature of the BF3 site in AR could be translatable to ER as well. The residues making up the pocket are more distinct in the ER compared to the other SHRs [326]. In ER, a helix in the hinge region folds onto the ER-LBD in the crystal, blocking the prospective BF3 region [89]. If the helix is removed from the potential BF3 pocket, the pocket is of similar structure and depth and is recognizable by MOE SiteFinder (Figure 19) [42]. Thus, future exploration of ER BF3 area is needed to deny or confirm druggability and functional relevance of that area.

## 4. Benchmarking ER Ligands

### 4.1. ER Decoy Datasets

One of the most widely used datasets for benchmarking CADD studies around ER are DUD and DUD-E [327,328]. Such datasets are important for training ER activity predictive models and for validation of docking experiments.

In 2006, Irwin et al. chose 40 target proteins to create DUD set with 36 decoys each generated from 2950 known ligands for these proteins as benchmarking sets for molecular docking including both ER agonists and antagonists [327]. ER target docking with DOCK3.5.54 was able to achieve an Enrichment Factor (EF) of ~100 for the top 98 molecules (which contained four known antagonists) [327,329]. In comparison with Rognan decoy dataset, Jain Decoys and MDDR decoys, the DUD dataset displayed inferior EF performance [327]. The authors attributed this to alleged easier decoys in the other datasets based on their physical properties such as variation in molecular weight category as seen in all the other decoys or lack of hydrogen binding properties as seen in Rognan’s decoys [327]. As mentioned above, Niinivehmas et al. used this dataset for their virtual screening pipeline [225]. In fact, Durrant et al. in 2015 used known ER agonists and antagonists from DUD and decoy compounds from NCI diversity set III to propose a novel neural network based scoring function to predict 39 novel ER ligands with the best hit, NCI-19136, reaching Ki value of 490 nM on ERα [330]. However, DUD has imbalanced net formal charge between ligands and decoys. The dataset also contains some false decoys that are confirmed binders due to shortcomings in property matching which could decrease the enrichment [331,332]. Vogel et al. and, Wallach and Lilien in 2011 tried to address this problem by building datasets DEKOIS (demanding evaluation kits for in silico screening) and Virtual decoy set (VDS), respectively [331,332].

Shoichet et al. (2012) extended the DUD database naming it DUD-E for 102 proteins, including ER with 50 decoys for each 22886 ligands [328]. They addressed the decoy and ligand similarity issue by using CACTVS fingerprints along with ECFP4 or daylight fingerprint for filtering out false decoys [328]. They also fitted the decoys to possible local chemical space of the ligands and added net charge was better solvation and electrostatic properties, which as seen in case of ER resulted in a higher log of the area under the receiver operating characteristic curve (AUC) value [328]. The improved property matching of molecular weight, hydrogen bond behavior, number of rotatable bonds, net charge and MiLogP between decoys and ligands was able to yield a better dataset with higher EF values than DUD [328]. As discussed above, Istyastono et al. (2012) and Pang et al. (2018) used the DUD-E database for virtual screening [229,238]. Moreover, in 2017, Istyastono and group used the DUD-E database for the validation of their three unbiased Virtual screening pipelines [333].

It should be noted that decoy datasets are prone to biases, such as “analog bias” that could arise due to the lack of variability in the decoys; “complexity bias” due to structural differences between the actives and decoys; and “false negative bias”, which is due to the mixing of active compounds into the decoys datasets resulting in undervaluation of models being evaluated [334]. Using highly filtered methods for selecting decoys and introducing true negatives into the datasets can help in combating these biases thus resulting is improved benchmarking datasets.

### 4.2. Endocrine Disruptor Program—ToxCast, Tox21 Datasets

Another marked benchmark dataset of ER ligands was generated within the EPA Endocrine Disruptors Program. It is a national initiative originated in 1996 when a Endocrine Disruptor Screening and Testing Advisory Committee was set up to come up with a consensus screening and testing protocol for red-flagging possible endocrine disruptors for the safety of American public under the Food Quality Protection Act [335].

A two-step approach is taken for the screening of potential endocrine disruptors in commercial chemicals, pesticides and environmental contaminants. Firstly, common chemicals that can potentially interact with the human endocrine system are identified. The EPA introduced ToxCast (Toxicity ForeCaster), a forecaster HTS method to prioritize compounds for screening based on concentration and bioactivity in cells or proteins that are exposed to the compounds using automated HTS [336,337]. The EPA also set up a joint initiative, Tox21 (Toxicology Testing in the 21st Century collaboration) program, with the NIH and the FDA which resulted in a larger library of compounds that are tested for their toxicity in humans [338]. This program was aimed to prioritize chemicals for further testing using automated screening through environmental compounds.

In 2014, Huang et al., as part of Tox21 initiative measured ER modulating activity (agonistic or antagonistic) for ~10000 compounds, using BG1 cells and ERα β-lactamase reporter assays in HEK293 cells [339]. In 2016, Huang et al. built a model for toxicity end point predictions using information from the SAR of 10,000 Tox21 compounds by in vitro tests at 15 concentrations to help prioritize compounds for testing [340]. Judson et al. in 2015 outlined and proposed a computational method to integrate results from 18 different in vitro HTS assays, including Tox21 and ToxCast that had been carried out for chemical activity on ER to prioritize chemicals for testing integrated for these different assays [341]. Secondly, the prioritized compounds generated from the model are then screened for their activity on estrogen, androgen and thyroid system to quantify the dose dependent adverse effect relationship [335]. After years of validating pipelines and protocols, as of 2015, the EPA has released a list of around 1800 compounds as potential endocrine disruptors for ER [342].

Subsequently, datasets accumulated from various sources, including the Endocrine Disruptor Program, were used in CERAPP (Collaborative ER Activity Prediction Project). CERAPP was a collaborative modeling initiative that resulted in 40 categorical and 8 continuous models from 17 research groups for prediction of ER activity of 32464 chemicals [342]. The models were trained on datasets from Tox21 collaboration and the ToxCast HTS data with about 1677 compounds categorized as ER agonists, antagonists or binders [342]. The prediction database was curated using 32,464 unique entries that humans could be exposed to from U.S. EPA chemical product categories database, Distributed Structure-Searchable Toxicity list, Canadian Domestic Substances list, Endocrine Disruption Screening Program and U.S. EPA’s Estimation Program Interface [342]. The experimental evaluation set consisted of 7522 unique entries for categorical models and 7253 for continuous models taken from Tox21, U.S. FDA Estrogenic Activity Database, Ministry of Economy, Trade and Industry (Japan) database and ChEMBL database [342].

The models were divided into three classes based on their prediction ability: 21 categorical and 3 continuous for binding models, 11 categorical and 3 continuous for agonist models and 8 categorical and 2 continuous for antagonist models [342]. A consensus pipeline was then set up to combine categorical model results into active or inactive and continuous models into very weak, weak, moderate or strong based on their activity for each class, followed by a consensus of many of these models with literature evaluation set taken from more than six sources and resulted in balanced accuracy of 0.91 [342]. The authors concluded that no QSAR or docking model could achieve 100% accuracy due to major issues in discrepancy in the literature and misclassification of very weak compounds due to not being tested in high enough concentrations [342]. In total, 4001 compounds were classified as actives and 28,463 as inactives which were further prioritized by activity using continuous consensus model [343].

Since then, many other researcher groups actively used Tox21, ToxCast and the CERAPP data in their CADD investigations. For instance, in 2016, Ribay and researchers from Rutgers University used the Tox21 dataset to train prediction model based on QSAR models to predict activity of compounds [344]. Ruiz et al. (2017) proposed consensus models based on the library of tier I endocrine disruptors from the US endocrine disruptor program and the training dataset from CERAPP to evaluate these datasets with the help of QSAR and docking for both AR and ER [345]. Similarly, Russo et al. in 2018, used datasets from ChEMBL, Pubchem and CERAPP (agonist and antagonist) to compare classic machine learning models such as AdaBoost, Bernoulli naive-Bayes, random forest, support vector classification and deep neural network models with varying layers, using five-fold validation [346]. They concluded that the random forest model performed the best over DNNs, thus suggesting that simpler machine learning algorithms are enough for ER binding predictions [346]. In the same year, Fernandez et al. developed a deep learning model on Tox21 dataset to predict compound toxicity for both AR and ER, based merely on molecular images [347]. In 2019, Francesco et al. created DeepDocking, a QSAR-based method allowing to predict docking scores for 1.36 billion ZINC15 molecules against ER-AF2 target site [348].

To summarize this section, we could postulate that further improvements in the structure-based CADD methods, pharmacophore-based models and larger training sets provided by large scale initiatives such as the Endocrine Disruptor Program and CERAPP initiative should further improve accuracy of ERα ligand predictions and could result in novel BCa drug candidates.

## 5. Future Perspective

SERMs and SERDs have been the main focus of drug development for ER for over 40 years, with all currently approved ER-directed drugs falling under these two categories. Research and development on SERMs and SERDs have cumulated but there is still room for improvement. In recent years, alternative chemotypes have been discovered that could result in subsequent generations of SERMs with higher potencies and less side effects [349]. However, long-term treatment with currently popular SERMs and SERDs may lead to development of resistance in BCa cells. Thus, alternative ER-targeting strategies have gained a momentum in recent years, as they may offer compounds that can overcome the resistance. Another way to approach this would be target alternative sites on the ER as we have recently attempted for AR [325,350,351,352,353,354,355].

The use of CADD approaches become invaluable support in such novel endeavors, among which the following might represent a particular promise:

1. Targeting the AF2 site: Blocking co-regulatory protein binding may potently inhibit co-regulator mediated ER transcriptional activity. This presents the most promising alternate site with a well-defined targetable cavity (Figure 14). The use of SBDD with a larger chemical database to screen through could result in diverse active chemotypes and lead compounds that have not yet been explored. Although significant progress has been made in AF2-site drug discovery in recent years, until now only the empirically designed ERX-11 has reached later stages of drug development pipeline, presenting a promising candidate for successful inhibition of BCa cells (Table 6) [287,288]. However, we are yet to see a small molecule inhibitor that has reached the clinical trial phase. Furthermore, screening protein–protein interaction libraries could also result in high hit rates as these libraries mimic protein interactions and have been shown to be advantageous over small molecule inhibitors for larger sites [356,357,358].
2. Targeting the F-region: Targeting this domain by enhancing the protein–protein interaction between the F-domain and 14-3-3 protein interaction interface could exert the desired selectivity in inhibition of ERα. Any molecule that enhances this interaction would increase the inhibitory effects of 14-3-3 on ERα. As discussed above, fusicoccin has shown inhibitory effects but displays very poor potency (Figure 18) [99]. Finding a potent protein–protein interface enhancer is challenging. Using a large chemical library with the help of DeepDocking would increase the chances of finding novel and diverse “molecular glue” molecules against this large binding site [348]. This strategy may very well help in alternative inhibition of ER activity through AF1 inhibition.
3. Targeting the potential ER-DBD P-box and D-box binding sites: Even though these two binding sites are potential for discovery of new inhibitors, it is challenging. Only a handful of inhibitors have been developed for targeting this domain with all focusing on the second zinc finger (Table 7). AA presents itself to be the strongest candidates of all but still display low potency, making it clinically unviable [310,312,313,314]. This site is challenging for both CADD and empirical-based approaches. Validation of these two sites for binding of small molecules is required to define a strong targetable site that could assist the CADD process. Furthermore, MD simulations may be performed on this dynamic system to find any targetable transient pockets on this domain [359,360,361].
4. Targeting ER-NTD: The NTD houses the important AF1 pocket that can result in hormone-independent transcriptional function of the ER. Targeting this pocket could help in total inhibition of ER activity. Since the region is highly elusive due to it intrinsically disordered nature in all of the SHRs, more structural biology efforts are needed to reveal structural details about the ER-NTD in order to define the small molecule binding pocket for rational SBDD. The combined use of FRAGFOLD-IDP and IDP-LZerD algorithm could be used to model these disordered stretches [47,48]. Once a viable structure has been predicted, binding site prediction and virtual screening could be employed in discovery of small molecule inhibitors that could target this site. However, in vitro and in vivo tests would still be necessary to prove the experimental value of these inhibitors. Given the uncertainty and the lack of viable structures of this domain, targeting the F-domain, as discussed above, would provide a more concrete strategy in inhibition of AF1 transcription.
5. Targeting ERα via selective dimerization inducers: This induced protein–protein interaction could be a promising approach for selective inhibition of ERα but more work needs to be done in this aspect. Since it is unclear how cosmosiin and angolesin induce selective dimerization of ER, to find potential novel molecules with the same nature, similarity search on these known compounds could be employed using tools such as ROCS (OpenEye) [362].

A drug discovery pipeline includes in silico screening along with in vitro and in vivo experiments for a balance between speed and quality [363]. As computational power and resources increase dramatically, the intelligent use of various CADD techniques that are already readily available to the scientific community should effectively accelerate ER-directed drug discovery.

In particular, the development of Deep-learning accelerated docking enables the virtual screening of billion compounds in ZINC 15 compound library thereby greatly expanding the chemical space that can now be explored [348]. Following the Best Practices of CADD, we have conducted extensive and rigorous research for discovery of target novel inhibitors on AF2 [350], BF3 [325,351,352] and DBD (both P-box [353,354] and D-box [355]) binding sites in AR as an alternative strategy to a similar conventional Androgen binding site driven inhibition for the same [38,363]. We have found potent inhibitors using computationally driven drug design pipelines and similar strategies can be applied to further development in small molecule inhibitors of ERα [38,363]. In fact, as discussed in Section 3.2, Kriti et al. used these practices in discovery of novel potent compounds against ER-AF2 [297,300]. Significant computational resources could be used to further explore and observe the effects of ER mutation and resistance and for drug discovery for these mutated proteins. In general, the rise of deep learning and artificial intelligence results in a newfound interest in approaching drug designing using novel CADD methodologies and is only set to increase in the coming years. However, it should always be kept in the focus that any in silico approach is not free from its own shortcomings, thus there will always be a need for high-quality in vivo and in vitro data, essential for creating any sound drug design pipeline.

## Figures and Tables

**Table 1:** List of notable Selective estrogen receptor modulators.

Number	Structure	Compound	Core
First Generation SERMs			
1	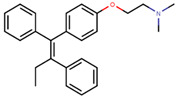	Tamoxifen	Triphenylethylene
2	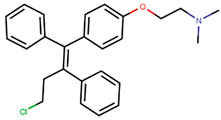	Toremifene	triphenylethylene
3	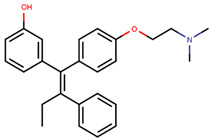	Droloxifene	triphenylethylene
4	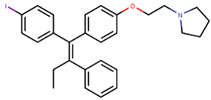	Idoxifene	triphenylethylene
Second Generation SERMs			
5	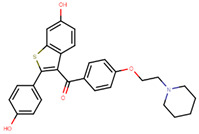	Raloxifene	Benzothiophene
Third Generation SERMs			
6	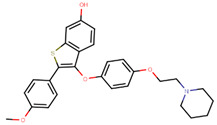	Arzoxifene	Benzothiophene
7	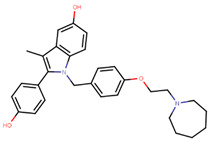	Bazedoxifene	Indole
8	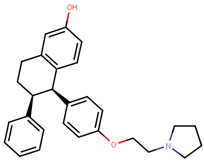	Lasofoxifene	Tetrahydronaphthalene
Fourth Generation SERM			
9	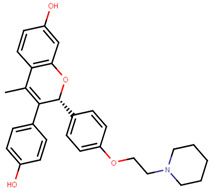	Acolbifene	benzopyran

**Table 2:** ER ligands validated with CADD.

Compound	Structure	Activity
Celecoxib	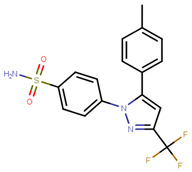	IC_50_ = 94.06 ± 14.03 µM (MCF-7 cell cytotoxicity assay)
S4	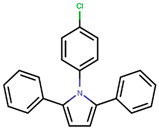	0.25 µM (FP assay)
YMA-005	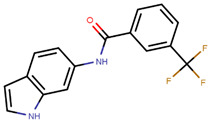	IC_50_ = 1.76 nM (ERα ELISA binding assays)
YMA-006	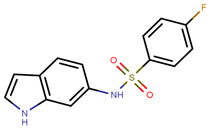	IC_50_ = 3.31 Nm (ERα ELISA binding assays)
19	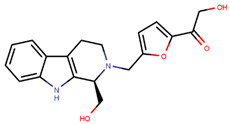	Only active at high Concentrations

**Table 3:** List of notable selective estrogen receptor degraders.

Compound	Structure	Company	Effects
Fulvestrant	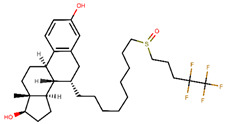	AstraZeneca	Development of resistance. Low bioavailability.
AZD9496	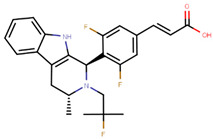	AstraZeneca	High Bioavailability. Cross-resistant to fulvestrant.
Elacestrant	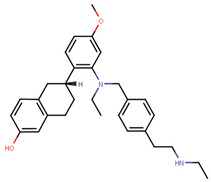	Radius Pharmaceuticals	High Bioavailability. Dose dependent SERM/SERD hybrid.
LSZ102	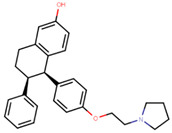	Novartis Pharmaceuticals	High Bioavailability.
GDC-0927	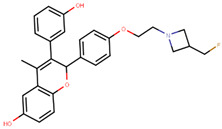	Seragon Pharmaceuticals/Genetec Inc	High Bioavailability. Highly active in tam-resistant cells.

**Table 4:** PROTAC ligands for ER.

Compound	Core (R1)	Linker (R2)	Degron (R3)
Protac-2	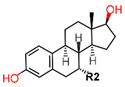	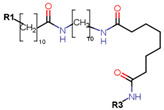	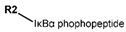
Protac-B	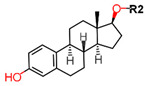	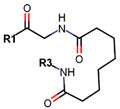	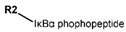
Compound 24	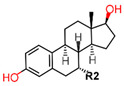	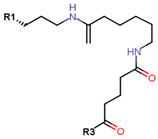	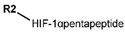
Compound 11	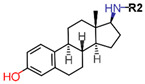	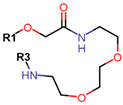	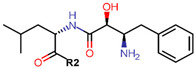
SNIPER(ER)-3	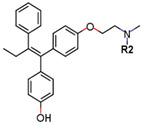	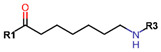	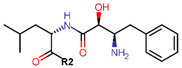
lipophilic amino acid Boc-Trp motif	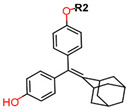	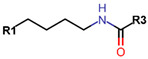	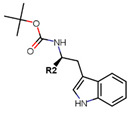
C3-linked adamantane motif	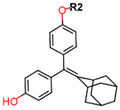	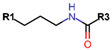	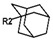
monocyclic trifluoromethyl cyclohexane motif	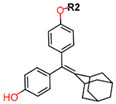	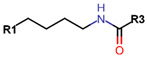	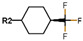
ERD-148	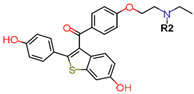	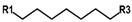	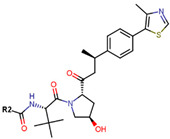
ERD-308	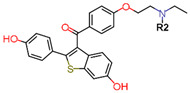	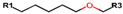	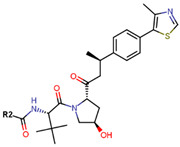

**Table 5:** Small molecule ER inhibitors, currently in clinical trials.

Compound	Structure	Clinical Trial ID and Phase	Mechanism of Action
Bazedoxifene	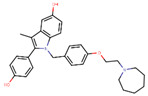	NCT02448771 (Phase Ib/II) [186] NCT02694809 (Phase II) [187]	SERM
Lasofoxifene	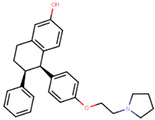	NCT03781063 (Phase II) [190]	SERM
Acolbifene	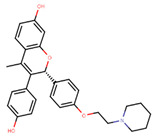	NCT00853996 (Phase II) [199]	SERM
AZD9496	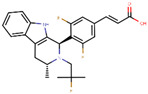	NCT03236974 (Phase I) [260] NCT02780713 (Phase I) [260] NCT02248090 (Phase I) [260]	SERD
Elacestrant	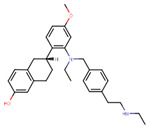	NCT03778931 (Phase III) [262]	SERD
LSZ102	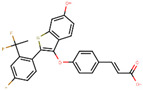	NCT02734615 (Phase I) [267]	SERD
GDC-0927	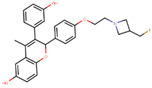	NCT02316509 (Phase I) [269]	SERD
H3B-5942	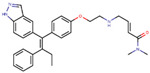	NCT03250676 (Phase I/II) [280]	SERCA

**Table 6:** AF-2 directed inhibitors.

Compound	Structure	Activity
12a	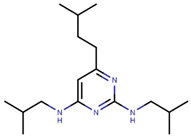	Ki = 29 µM (FAA) Ki = 0.91 (TR-FRET)
18	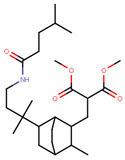	Ki = 7.1 µM (TR-FRET)
11c	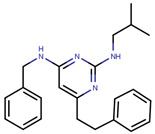	Ki = 1.7 μM (TR-FRET) Ki = 1.9 μM (Reporter Gene Assay)
3c	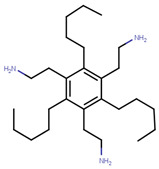	Ki = 1.7 μM (TR-FRET) Ki = 3.2 μM (Reporter Gene Assay) Ki = 3.2 μM (M2H Assay)
3d	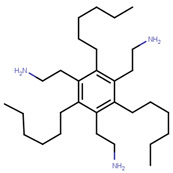	Ki = 2.0 μM (TR-FRET) Ki = 3.6 μM (Reporter Gene Assay) Ki = 3.4 μM (M2H Assay)
3e	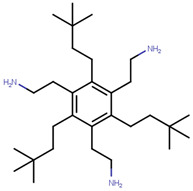	Ki = 2.1 μM (TR-FRET) Ki = 3.8 μM (Reporter Gene Assay) Ki = 2.2 μM (M2H Assay)
1g	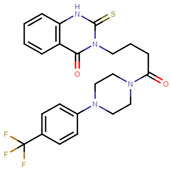	IC_50_ = 2.3 μM (Reporter Gene Assay)
7	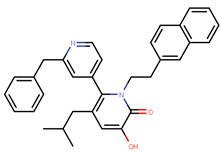	Ki = 4.2 μM (FP assay)
ERI-05	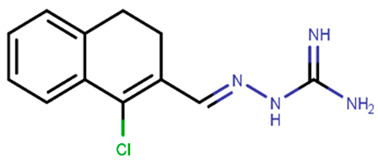	IC_50_ = 5.5 μM (M2H assay, COS7 cells) IC_50_ = 3.6 ± 0.6 μM (M2H assay, HEC-1 cells) IC_50_ = 1.2 ± 0.1 μM (Reporter gene assay)
20	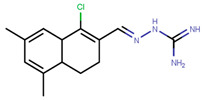	IC_50_ = 1.2 ± 0.1 μM (Reporter gene assay) IC_50_ = 2.5 ± 1.1 μM (M2H assay, HEC-1 cells)
22	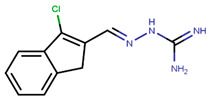	IC_50_ = 1.3 ± 0.1 μM (Reporter gene assay) IC_50_ = 6.8 μM (M2H assay, HEC-1 cells)
29	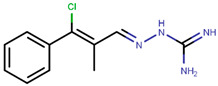	IC_50_ = 1.4 ± 0.4 μM (Reporter gene assay) IC_50_ = 2.6 μM (M2H assay, HEC-1 cells)
VPC-16230	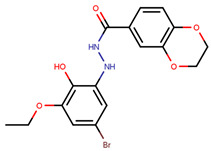	IC_50_ = 2.98 μM, (TR-FRET)
VPC-16464	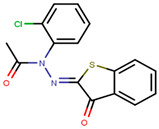	IC_50_ = 2.7 μM, (TR-FRET)
VPC-16606	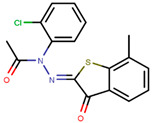	IC_50_ = 0.3 μM, (TR-FRET)
ERX-11	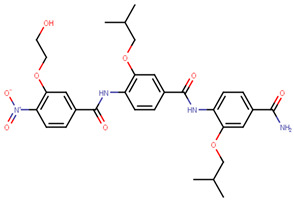	IC_50_ = 250–500 nM

**Table 7:** DNA Binding Domain targeting compounds.

Compound	Structure	Mechanism
DIBA	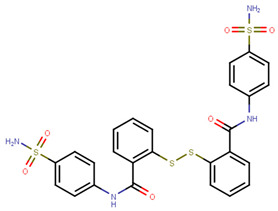	Zinc Chelator
BITA	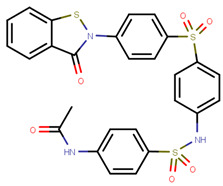	Zinc Chelator
15d-PGJ2	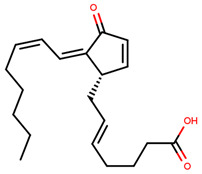	Direct covalent modifications of Cys227 and Cys240
TPBM	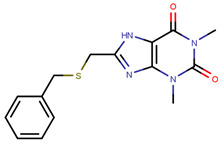	Unknown
AA	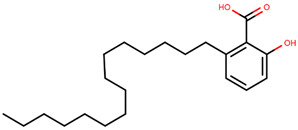	Inhibits ER-DBD/ERE binding

## Abbreviations

BCa	Breast cancer
ER	Estrogen Receptor
EU	European Union
PR	Progesterone Receptor
HER2	Human epidermal growth factor 2
HR	Hormone receptor
SERM	Selective estrogen receptor modulator
SERD	Selective estrogen receptor degrader
HT	Hormone therapy
AI	Aromatic inhibitors
HTS	High throughput screening
CADD	Computer-aided drug design
SBDD	Structure based drug design
IDP	Intrinsically disordered proteins
MD	Molecular dynamics
SAR	Structure–activity relationship
EPA	Environmental protection agency
TF	Transcription factor
SHR	Steroid Hormone receptor
AR	Androgen receptor
GR	Glucocorticoid receptor
MR	Mineralocorticoid receptor
NTD	N-terminal domain
DBD	DNA binding domain
LBD	Ligand binding domain
AF1	Activation function-1
ERE	Estrogen response element
AF2	Activation function-2
EBS	Estrogen binding site
E1	Estrone
E2	17β-Estradiol
E3	Estriol
E4	Esterol
OHT	4-hydroxy-tamoxifen
VS	Virtual screening
PLIF	protein–ligand interaction fingerprints
Tc	Tanimoto coefficient
DUD	directory of useful decoys
FP	Fluorescence polarization
DUD-E	DUD enhanced
PROTAC	Proteolysis-targeting chimeras
VHL	Von Hippel-Lindau
SNIPERs	Specific and nongenetic IAPs-dependent protein erasers
cIAP1	Cellular inhibitor of apoptosis protein 1
TD	N-terminal aspartic acid cross linking
TD-PERM	TD peptidomimetic ER modulator
SERCA	Selective ER covalent antagonist
ERX-11	ER co-regulator binding modulator-11
FAA	Fluorescence anistropy assay
TR-FRET	Time-resolved fluorescence resonance energy transfer
M2H	Mammalian two-hybrid
DIBA	2,2′- dithiobisbenzamidine
BITA	Benzisothiazolone
15d-PGJ2	15-Deoxy-Delta-12,14-prostaglandin J2
TPBM	8-benzylsulfanylmethyl-1,3-dimethyl-3,7-dihydropurine-2,6-dione
AA	Anacardic acid
BRET	bioluminescence resonance energy transfer
BF3	Binding function -3
EF	Enrichment factor
DEKOIS	demanding evaluation kits for in silico screening
VDS	Virtual decoy set
AUC	Area under the receiver operating characteristic curve
ToxCast	Toxicity ForeCaster
Tox21	Toxicology Testing in the 21st Century collaboration
CERAPP	Collaborative ER Activity Prediction Project
